# The role of ubiquitin ligase E3A in polarized contact guidance and rescue strategies in UBE3A-deficient hippocampal neurons

**DOI:** 10.1186/s13229-019-0293-1

**Published:** 2019-11-29

**Authors:** Ilaria Tonazzini, Geeske M. Van Woerden, Cecilia Masciullo, Edwin J. Mientjes, Ype Elgersma, Marco Cecchini

**Affiliations:** 1 Istituto Nanoscienze- Consiglio Nazionale delle Ricerche (CNR) & Scuola Normale Superiore, NEST, Piazza San Silvestro 12, 56127 Pisa, Italy; 2 Department of Neuroscience, ENCORE Expertise Center for Neurodevelopmental Disorders, Erasmus MC, Wytemaweg 80, 3000 CA Rotterdam, the Netherlands

**Keywords:** Ubiquitin ligase E3a (UBE3A), Contact guidance, Angelman syndrome, Axonal guidance, 15q duplication autism, Nocodazole, Microgratings, Cytoskeleton

## Abstract

**Background:**

Although neuronal extracellular sensing is emerging as crucial for brain wiring and therefore plasticity, little is known about these processes in neurodevelopmental disorders. Ubiquitin protein ligase E3A (UBE3A) plays a key role in neurodevelopment. Lack of UBE3A leads to Angelman syndrome (AS), while its increase is among the most prevalent genetic causes of autism (e.g., Dup15q syndrome). By using microstructured substrates that can induce specific directional stimuli in cells, we previously found deficient topographical contact guidance in AS neurons, which was linked to a dysregulated activation of the focal adhesion pathway.

**Methods:**

Here, we study axon and dendrite contact guidance and neuronal morphological features of wild-type, AS, and UBE3A-overexpressing neurons (Dup15q autism model) on micrograting substrates, with the aim to clarify the role of UBE3A in neuronal guidance.

**Results:**

We found that loss of axonal contact guidance is specific for AS neurons while UBE3A overexpression does not affect neuronal directional polarization along microgratings. Deficits at the level of axonal branching, growth cone orientation and actin fiber content, focal adhesion (FA) effectors, and actin fiber–binding proteins were observed in AS neurons. We tested different rescue strategies for restoring correct topographical guidance in AS neurons on microgratings, by either UBE3A protein re-expression or by pharmacological treatments acting on cytoskeleton contractility. Nocodazole, a drug that depolymerizes microtubules and increases cell contractility, rescued AS axonal alignment to the gratings by partially restoring focal adhesion pathway activation. Surprisingly, UBE3A re-expression only resulted in partial rescue of the phenotype.

**Conclusions:**

We identified a specific in vitro deficit in axonal topographical guidance due selectively to the loss of UBE3A, and we further demonstrate that this defective guidance can be rescued to a certain extent by pharmacological or genetic treatment strategies. Overall, cytoskeleton dynamics emerge as important partners in UBE3A-mediated contact guidance responses. These results support the view that UBE3A-related deficits in early neuronal morphogenesis may lead to defective neuronal connectivity and plasticity.

## Introduction

In the brain, neurons are embedded in a dense environment, the extracellular matrix (ECM), which contains a complex array of directional cues. Neuronal development and migration are governed by molecular stimuli, acting over long distances, and by physical signals locally retrieved through contact guidance [1, 2]. Here, contact sensing triggers complex intracellular signaling patterns that are integrated by cells to guide neuronal adhesion, migration, neurite wiring, and synaptic plasticity [2, 3]. The outgrowth of neurites in neuronal cells is critically controlled by the development of focal adhesions (FAs), both in vitro [4, 5] and in vivo [6, 7]. FAs act as sensors by integrating signals from both the ECM and chemotactic factors [3, 8] and mediate coordinated rearrangements of the cytoskeleton, essential for both neuronal growth and synaptic functionality [7]. Neurons integrate information from multiple sources at the level of cytoskeletal signalling that ultimately modulates cell shaping, migration, and contractility [9]. Thereafter, neuronal growth and guidance to the proper targets require concerted efforts from the cytoskeleton (actin and microtubule) and from adhesions [10]. In this framework, the dilated tip of developing neurites, i.e., the growth cones (GCs), sense environmental cues leading the axons to their specific targets for precise neuronal wiring. The *actin* cytoskeleton is the major component of the GC that powers its directional motility [10]. However, *microtubules* are also important in neuronal/growth cone guidance, because their polarized invasion into the peripheral domain on one side of the GC is essential for it to turn [11].

Deficits in neuronal micro-connectivity leading to functional connectivity deficits are recently emerging as crucial in many cognitive disorders (e.g., autism spectrum disorders, schizophrenia) [12]. However, the role of neuronal sensing mechanisms during development and migration is under-investigated in pathological conditions. Recently, a pivotal role of ubiquitination (i.e., at the level of E3 ligases) emerged in the processes which orchestrate adhesion and cytoskeletal signaling pathways [13]. Among these, the ubiquitin protein ligase E3A (UBE3A) has a key role in neurodevelopment, in particular at early neurodevelopmental stages [14]. Importantly, the exact level of UBE3A in the brain is crucial: its lack leads to Angelman syndrome (AS; OMIN#105830) [15], while its increase can cause non-syndromic autism spectrum disorder (ASD) and Dup15q syndrome (OMIN#608636) [16–18]. AS and Dup15q show phenotypic overlap characterized by autistic features, intellectual disability, motor deficits, speech absence/delay, and epileptic seizures [19, 20]. A strong correlation between AS-associated deficits and the loss of UBE3A ligase activity has been reported [21], as well as between an autism UBE3A-linked mutation and the hyper-activation of UBE3A [22]. Importantly, the *Ube3a* gene is transcribed to form distinct splice variants encoding two UBE3A protein isoforms (designated as isoforms 2 and 3, in mice). Recent studies showed that these isoforms have different cellular localization and likely different function [23–25].

*Ube3a*-deficient mouse models showed abnormalities at the level of dendritic arborization and spine development (both in vitro and in vivo), even if with overall conflicting outcomes [23, 24, 26–30], as well as at the level of spine actin reorganization [31, 32] and dendritic polarity in vivo [23]. In addition, the increase of UBE3A leads to a reduction in dendritic arborization and synapse formation in cortical neurons [30]. Finally, UBE3A has been reported to be highly present at GCs [26, 33]. Although several UBE3A targets were described [34, 35], still little is known about the role of UBE3As in neuron morphogenesis and the pathogenesis of UBE3A-associated disorders. However, it is noteworthy that UBE3A gene reinstatement in adult mice rescued electrophysiological properties but did not result in a behavioral rescue, suggesting that UBE3A could be involved in neuronal wiring [14, 36].

Recent developments in biomaterial science allow direct investigation of the processes that control cell contact sensing by using nano-textured substrates [37]. Nano/microstructured surfaces are capable of tuning the properties of the cell surroundings at the nanoscale and to test cell response to physical stimuli in vitro [38, 39]. Microgratings (GRs; anisotropic topographies composed of alternating lines of grooves and ridges with sub-micrometer lateral dimensions) could effectively apply directional topographical stimuli via pure contact interaction, and tune neuronal differentiation, polarization, and neurite orientation [40–43]. We previously demonstrated, in differentiating PC12 neuronal cells on GRs, that contact guidance requires Rho-associated protein kinase (ROCK)-myosin-II-activated contractility [4, 44] and dynamically responds to topographical noise [45]. Here, nocodazole treatment was demonstrated to improve the neurite alignment to the GR pattern when the topographical stimulus was disturbed [45]. Importantly, thanks to GRs, we showed for the first time the aberrant morphological phenotype of AS neurons in vitro: neurite contact guidance is defective in *Ube3a*-deficient (i.e., model of AS) hippocampal neurons at early stages of development (DIV1-3), and this phenotype is linked to an impaired activation of the FA signaling pathway (at the level of FAK phosphorylation) [5].

Here, we selectively study axon and dendrite topographical guidance of wild-type, UBE3A-deficient, and UBE3A-overexpressing neurons on micro-grooved substrates. The aim is to unravel the role of UBE3A in neuronal contact guidance and explore neuronal morphological aspects relevant to AS and ASD/Dup15q syndrome. We exploit GRs (with a pattern of 1-μm ridge, 1-μm groove, and 500-nm depth), which transfer an optimal directional impulse to the cells. We further investigate the FA molecular pathway and the link between FA and actin fibers, at the level of α-actinin organization. Finally, we test the potential of either UBE3A reinstatement or pharmacological treatment acting on cytoskeletal contractility for restoring correct topographical guidance in UBE3A-deficient neurons.

## Methods

### Substrate fabrication

The topography with GRs (lines of micron ridges and grooves) was fabricated by means of electron-beam lithography (EBL) and reactive ion etching (RIE) starting from a commercial p-doped silicon wafer (SYLTRONIX, France), as previously reported [45, 46]. Perfluoropolyether (PFPE) resin (FLUOROLINK® MD 700, Solvay Specialty Polymers, Bollate, Italy) was mixed with 3% w/w photoinitiator Darocur 1173 (C10H12O2, 405655 Sigma Aldrich), poured on top of the GR initial silicon mold, and crosslinked by UV-light (365 nm, 25 mW cm^−2^) as reported in [47]. Cyclic olefin copolymer (COC) foils (thickness of 140 μm, Microfluidic ChipShop GmbH, Jena, Germany) were imprinted using an Obducat Nanoimprint 24 system (Obducat, Lund, Sweden) using the PFPE molds. COC foils were softened by raising the temperature up to 150 °C, then a pressure of 50 bar was applied for 300 s, before cooling down to 70 °C (i.e., below the glass transition temperature of the copolymer—*T*_g_ = 134 °C). Finally, the pressure was released and the mold was detached from the imprinted COC with a scalpel [48]. The imprinted substrates were quality checked by optical microscopy and attached to the bottom of hollowed 35-mm Petri dishes by using silicone glue (RS Components RS692-524). Before cell culturing, the samples were sterilized by treatment with ethanol and then rinsed with H_2_O_mQ_ twice. GRs were fabricated with a pattern of 1-μm ridge and 1-μm groove width (period 2 μm) and 500-nm depth. Some COC foils were imprinted with no pattern to have flat substrates (with the same physic-chemical characteristics of GRs) and used as reference substrates (Flat).

### Mice

Mice were housed in individually ventilated cages (IVC; 1145 T cages from Techniplast) in a barrier facility. All animals were kept at 22 ± 2 °C with a 12-h dark and light cycle. All animal experiments were conducted in accordance with the European Commission Council Directive 2010/63/EU (CCD approval AVD101002016791 to Elgersma lab). Ube3a^m+/p–^ females [15] were crossed with wild-type males (all mice in 129S2 background), and hippocampal neuronal cultures were prepared from the brains of single (E16.5-P0) pups, which were either wild-type (WT) or Ube3a^m-/p+^ (designated as AS). Pregnant dams were sacrificed by cervical dislocation, while embryos were decapitated with surgical scissors. From each pup, a piece of tail tissue was used for genotyping, as in [15].

### Primary hippocampal cultures

Mouse hippocampal neurons (HNs) were isolated from male and female WT/AS brains (E16.5-P0) and prepared as previously described [5]. In brief, the hippocampi were removed from the brain, collected in 1 ml of cell medium on ice, washed two times with medium, and incubated in trypsin/EDTA solution (Thermo Fisher, Waltham, Massachusetts, USA), at 37 °C for 20 min. After another wash, the cells were suspended in neurobasal medium (NB) supplemented with 2% B27, 1% penicillin/streptomycin, and 1% glutamax (Thermo Fisher) and dissociated using a gently flamed Pasteur pipette. HNs were plated on poly-L-lysine-coated (37.5 ug/ml; P4832 Sigma-Aldrich, St. Louis, MO, USA) and laminin-coated (2.5 ug/ml, L2020 Sigma-Aldrich; in 0.1 M borate buffer pH 8.5) GR or flat substrates at a density of 30,000 cells/cm^2^, in a drop in 35-mm dishes. After 3 h, additional NB supplemented with 2% B27, 1% penicillin/streptomycin, and 1% glutamax were added to dishes (final volume 1 ml). For selected experiments only, we also established HNs from mixed WT and AS animals (see Additional file 1: Figure S1).

### Transfections

For UBE3A over-expression or reinstatement conditions (WT+UBE3A or AS+UBE3A, respectively), WT or AS HNs were plated on GRs and allowed to adhere for 32–40 h (early DIV2), then were transfected with a dual expression vector (pYE722) expressing both the mouse UBE3A isoforms 2 and 3 in addition to tdTomato (developed by the Elgersma lab; for details see Additional file 1: Figure S2). The control samples were transfected with an empty tdTomato-expressing plasmid. Plasmid DNA was extracted with a Qiagen Plasmid Kit (Qiagen, Hilden, Germany), according to kit instructions. Plasmid DNA concentration and purity were measured with a biophotometer, followed by storage at – 20 °C.

For each sample (size = 12-well plate), the transfection solution was prepared by adding 2.5 μg plasmid DNA and 3.3 μl Lipofectamine 2000 (ThermoFisher) in 200 μl NB. After 5 min of incubation at room temperature, the transfection solution was added dropwise to the neurons in 1 ml fresh NB supplemented with L-glutamine. After 45 min at 37 °C in cell incubator, HN samples were rinsed in warm NB and finally incubated back with their original conditioned medium (previously stored in a different plate). Two days after transfection (DIV4), HNs were fixed and processed for immunocytochemistry and imaging (see below).

### Cell pharmacological treatments

For contractility modulation experiments, WT and AS HNs were allowed to grow for 32–40 h (early DIV2) then were treated with nocodazole (methyl-[5-(2-thienylcarbonyl)-1H-benzimidazol-2-yl]-carbamate, dissolved in DMSO, 40 nM; *Noco*) or blebbistatin (1-phenyl-1,2,3,4-tetrahydro-4-hydroxpyrrolo[2,3-b]-7-methylquinolin-4-one, dissolved in DMSO, 25 μM; ***Bleb***); DMSO concentration never exceeded 0.2% v/v, and the corresponding solvent concentration was added to the untreated control condition. Noco was used to increase cell contractility via RhoA-ROCK activation (thanks to its microtubule destabilizing activity leading to GEF-H1 release) [49], while Bleb, an inhibitor of myosin II, to decrease cell contractility [4]. HNs were cultured up to DIV4: at DIV4, HNs were freshly treated with Noco or Bleb for 1 h and then were processed for immunostaining or cell lysis.

### Neuron immunocytochemistry

Control/transfected/treated HNs were cultured on GRs up to DIV4, fixed for 15 min with 4% formaldehyde with 4% sucrose in PBS at room temperature and processed as previously reported [5]. For axon-dendrite staining, the cells were incubated with primary antibodies anti-SMI312 (mouse, 1:100; Abcam ab24574), as axonal marker, and anti-MAP2 (guinea pig, 1:500; Synaptic Systems 188004), as dendrite marker; for some samples, anti-Tau antibody (rabbit, 1:1000; Synaptic Systems 314002) was used as axonal marker. Samples were incubated in GDB buffer (0.2% BSA, 0.8 M NaCl, 0.5% Triton X-100, 30 mM phosphate buffer, pH 7.4) overnight at 4 °C and then with appropriate secondary antibodies conjugated to AlexaFluor-488 and 647 (Invitrogen, 1:150) respectively in GDB at room temperature (for 1 h). For GCs staining, HNs were incubated with mouse anti-Tubulin-α (Sigma T5168; 1:500) primary antibody together with phalloidin-AlexaFluor647 (Invitrogen A22287; 1:40). For selected experiments, to check the UBE3A expression levels in WT+UBE3A or AS+UBE3A samples, WT/AS+UBE3A HNs were fixed and processed for immunostaining with anti-UBE3A (1:500; Sigma-Aldrich E8655) and anti-MAP2 antibodies in GDB buffer and then with the appropriate secondary antibodies conjugated to AlexaFluor488 and 647, respectively (Additional file 1: Figure S2a). After final washing in PBS and H_2_O_mQ_, samples were mounted using Fluoroshield mounting medium with 4′,6-diamidin-2-fenilindolo (DAPI) to stain nuclei (Sigma, F6057).

### Fluorescence confocal microscopy

Confocal images were acquired using a laser scanning confocal microscope TCS SP2 (Leica Microsystems, Wetzlar, Germany) equipped with × 40 or × 63 oil objectives, an argon (488, 561, and 633 nm) laser, and a 375-nm laser. All microscope settings were kept constant during sample imaging. Each confocal image (1024 × 1024 pixel resolution) was obtained from a *z*-series (stack depth was within 10 μm; steps = 1 μm; each image was averaged three times) and was chosen to cover the entire region of interest from top to bottom. The resulting *z*-stack was processed by ImageJ software into a single image using “z-project” and “max intensity” options. The confocal settings were kept the same for all scans when fluorescence intensity was compared.

### Axon-dendrite analysis

Neuronal morphometric data were collected using ImageJ (National Institute of Health, Bethesda, MD, USA). The GR direction was measured as an angle by the ImageJ Angle tool (for flat substrates, a randomly chosen direction was used).

Only neurons fully visible and with soma and neurites not in close contact with neighboring cells were traced and analyzed. SMI312 and MAP2 images were merged, and for each neuron, a *single axon* was chosen as the longest and SMI312 positive neurite [50]. Axons were semi-automatically traced (from the point of origin at the perimeter of the cell body to the tip of the growth cone) by NeuronJ (a plugin of ImageJ). Axonal secondary branching (SMI312-positive), if present, and dendrites (MAP2-positive) were semi-automatically segmented by NeuronJ according to their branching, as in [51]. A file containing the axonal or dendritic tracks were exported and loaded in Matlab (MathWorks, Natick, MA, USA) where a custom program, as in [5], calculated their *alignment* (measured by approximating the neurite segment as a straight line from the initial to end point and taking the angle of this line versus the GR orientation; in °); *length* (the distance of the traced segment path, in μm); and *straightness* (ratio between the distance from the initial and end point of the axon/dendrite and its length), as in [5]; a minimum threshold length of 10 μm was imposed for this analysis. For each axon, the mean axonal diameter (in μm) was measured, in its distal part (> 25 μm from the soma). We also measured the amount of axonal branches/neuron (in μm), their mean alignment angle, and the percentage of neurons with (main) branches in the axon (over the total number of neurons analyzed). Finally, we registered the *total neuritic network* and *total dendritic tree* developed by each neuron analyzed (in μm). At least 10 or 15 neurons were analyzed for each sample, in different conditions; the details are reported in each figure legend.

As first, we measured and collected neuronal morphometric data separately from WT/AS control HNs transfected with Tomato empty plasmid and from WT/AS control HNs not transfected but treated with DMSO 0.2% (for pharmacological treatments experiments). The results of morphological measurements showed that there are no differences between the two control conditions (see Additional file 1: Figure S4a), so data for axon-dendrite analysis from WT and AS control samples of both cell transfection and pharmacological treatment experiments were merged together (WT and AS).

Moreover, the MAP2 images were used to evaluate the cell *soma* morphological characteristics by ImageJ, as in [41]. Cell soma contours were drawn by the “Free-hand selection” tool and processed by the “Measurement” tool (with the options “Area,” “Fit ellipse,” and “Feret’s diameter”). The orientation of the GR patterns was measured by the “Angle tool” of ImageJ. The parameters measured in this analysis were soma area (μm^2^); soma major axis and minor axis for the best-fitted ellipse of the cell soma [52]; and soma alignment angle (angles were calculated as the absolute value of the difference between the orientation angle of the GRs and of the cell major axis).

### Growth cones analysis

The confocal images of actin fiber by phalloidin staining were used to evaluate GC morphology by ImageJ. GC contours were drawn by the “Free-hand selection” tool and processed by the “Measurement” tool (options Feret’s diameter, Mean gray value, and Area). For each GC ROI, the parameters measured in this analysis were GC actin mean intensity (a.u.); GC alignment angle (angles were calculated as the absolute value of the difference between the orientation angle of the GR and that of the GC major axis-F); GC area (μm^2^). GCs were considered *aligned* (//) to the GRs if the alignment angle was between 0° and 30° and *perpendicular* (┴) if the latter was between 60° and 90°; the amount of // or ┴ GCs was reported as the percentage over the total number. At least 40 GCs were analyzed per sample. Similar to ref. [42], the GC actin content was quantified by measuring the neat intensity of the fluorescent phalloidin signal: data were reported as the ratio of the fluorescence intensity from aligned GCs over the fluorescence intensity from the perpendicular ones.

### α-Actinin transfection and analysis

HNs were plated on GRs (or on flat surfaces for control experiments) and then were transfected (DIV5-6) with α-actinin-red fluorescent protein (RFP) vector (present from Biolab Technology AG, Zürich, Swiss) by Lipofectamine 2000 (as reported above). Two days after transfection (DIV8), HNs were fixed, mounted, and processed for imaging (as above).

α-Actinin organization was quantified by analyzing the α-actinin fluorescence signal of each HN by the Directionality tool of the software Fiji (http://fiji.sc/Fiji), similar to [42]. This plugin returned a directionality histogram by exploiting image fast Fourier transform (FFT) algorithms: isotropic structures generate a flat histogram, whereas oriented ones give a peaked histogram. These histograms were finally fitted by Gaussian curves that returned *directionality* (the center of the Gaussian curve), representing the direction in which it is oriented (here normalized to the GR pattern orientation direction). We analyzed at least 10 neurons/sample; image dimensions were kept fixed to 375 × 375 μm^2^.

The spatial distribution of the α-actinin signal was analyzed by the “Plot profile” option in ImageJ: this tool displays a two-dimensional graph of the intensities of pixels along a rectangular selection. For each cell, a ROI encompassing the neuronal soma and the initial segments of neuronal branching (mean area ± SD = 250 ± 64 μm^2^) was selected on the α-actinin image and on the relative bright field image of the underlying GRs. Images were processed by the Plot profile, and the resulting data were analyzed by FFT in Origin software (OriginLab, Northampton, MA). For each cell soma, we quantified the amplitude of the α-actinin signal at the frequency of the GR pick (e.g., GR periodic structure gives a frequency pick at 0.5), such as measurement of its bundle organization and periodicity. This value was then normalized to the correspondent α-actinin fluorescent signal (i.e., the RFP mean intensity value, calculated on the same ROI), to avoid any influence of different transfection levels, and reported as *α-actinin periodicity* (a.u.).

### Western blot and focal adhesion pathway activation

Western blot analysis on HNs (DIV4), under basal or Noco-/Bleb-treated conditions, was performed to assess the presence and activation (phosphorylation) levels of effector proteins in FA pathway, such as focal adhesion kinase (FAK), proto-oncogene tyrosine-protein kinase Src (SRC), talin, zyxin, and vinculin. WT and AS HNs were cultured on standard 12-well plates for 4 days and lysed on ice in RIPA buffer (Sigma-Aldrich, R0278) containing protease and phosphatase inhibitors cocktail (cOmplete and PhosSTOP, Roche Diagnostics, Basel, Switzerland). Cell lysates were centrifuged (15,000*g* for 25 min, 4 °C), and then the supernatants were tested for protein concentration by a protein assay kit (Micro BCA™, Thermo Scientific Pierce). The samples were mixed with Laemmli buffer containing β-mercapto-ethanol (5% final concentration), boiled for 5 min and used for gel electrophoresis (or kept at − 80 °C). HN lysates (10 μg/lane) were processed by immunoblot, as in [5]. Briefly, samples were resolved by gel electrophoresis (SDS-PAGE) using Gel Criterion XT-Precast polyacrylamide gel 4-12% Bis-Tris (Biorad), transferred to nitrocellulose membranes and probed overnight at 4 °C with primary antibodies. We used the following antibodies against: FAK (1:750; Abcam, Cambridge, UK; ab40794) and phospho(Tyr397)-FAK (1:1000; Abcam ab4803); SRC-(pan) (1:1000; Cell Signaling #2123); phospho(Tyr416)-SRC (1:1000; Cell Signaling #2101); talin-pan 1-2 (1:1000; Sigma clone 8D4); zyxin (1:1000; Abcam Ab71842); vinculin (1:1000; Abcam ab18058); actin (1:2500; Sigma-Aldrich A-3853). Membranes, after incubation with the appropriate peroxidase-linked secondary antibodies (goat anti-Rabbit/Mouse IgG-HRP Conjugate, Biorad; 1:2500) were developed by the SuperSignal West Femto (Thermo Scientific Pierce, #34095) or ClarityTM (Biorad, 170-5060) enhanced chemiluminescent (ECL) substrates. The chemiluminescent signal was acquired by ImageQUANT LAS400 scanner (GE Healthcare Life Sciences, Uppsala, Sweden). The density of immunoreactive bands was quantified by ImageJ; the results for pFAK and pSRC were normalized to the total FAK or SRC protein levels while for other proteins to the actin content, and reported in % in respect to WT levels.

### Statistical analysis

Data are reported as average value ± the standard error of the mean (mean ± SEM), if not differently stated. All the experiments were repeated at least three times independently for each reported dataset (*n* ≥ 3 independent samples for each condition), if not differently stated. Data were statistically analyzed by GraphPad PRISM 5.00 program (GraphPad Software, San Diego, CA, USA). For parametric data (the mean values obtained in each repeated experiment were assumed to be normally distributed about the true mean), Student’s *t* test (unpaired, two-tailed) or one-way ANOVA (Bonferroni’s multiple comparison test) analysis were used, if not differently stated; for selected comparisons between untreated/treated samples, Bonferroni’s selected tests were also used. Statistical significance refers to results where *P* < 0.05 was obtained.

## Results

### UBE3A unbalances role in axon and dendrite contact guidance

**Fig. 1:**
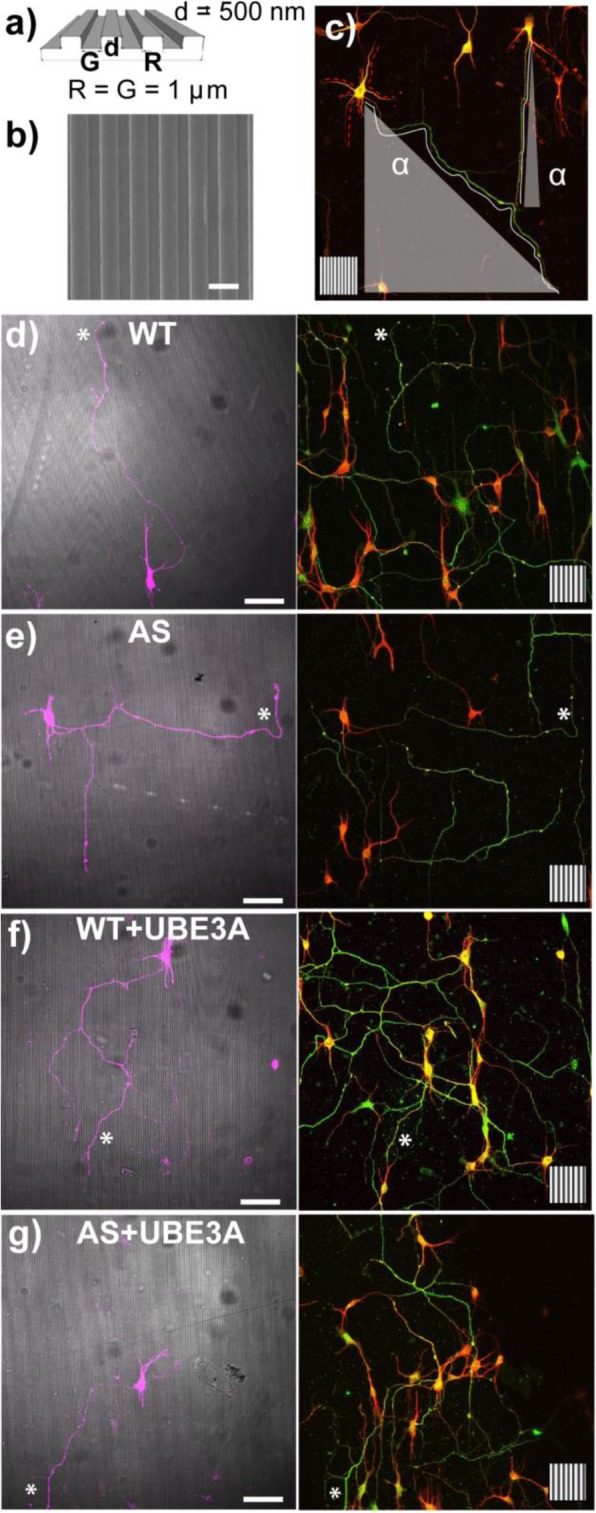
**a** Scheme of micrograting (GR) substrates: the pattern has 1-μm ridge (R) and 1-μm groove (G) lines (lateral period = 2 μm; T2) and GR depth (*d*) = 500 nm. **b** Scanning electron microscopy image of thermoplastic GRs (scale bar = 1 μm). **c** Scheme of axon/dendrite tracings for 2 neurons: axon = white continuous line, starting from cell soma; dendrites = dotted red lines; the alignment of each neurite was calculated as the alignment angle **α** between each neuritic trace (here outlined only for axons such as example) and the underlying GR direction (showed as inset, side 50 μm). **d**–**g** Representative confocal images of WT (**d**), AS (**e**), WT+UBE3A (**f**), and AS+UBE3A (**g**) HNs. WT and AS neurons were cultured on GRs, transfected (at early DIV2) with empty-Tomato vector (**d**–**e**) or with UBE3A 2/3-Tomato vector (**f**–**g**) (WT+UBE3A, model of UBE3A overexpression) and immunostained for axonal marker (SMI312, green) and dendrite marker (MAP2, red) (at DIV4); the asterisk indicates axons; the underlying GR pattern is reported as inset (scale bars = 50 μm)

**Fig. 2:**
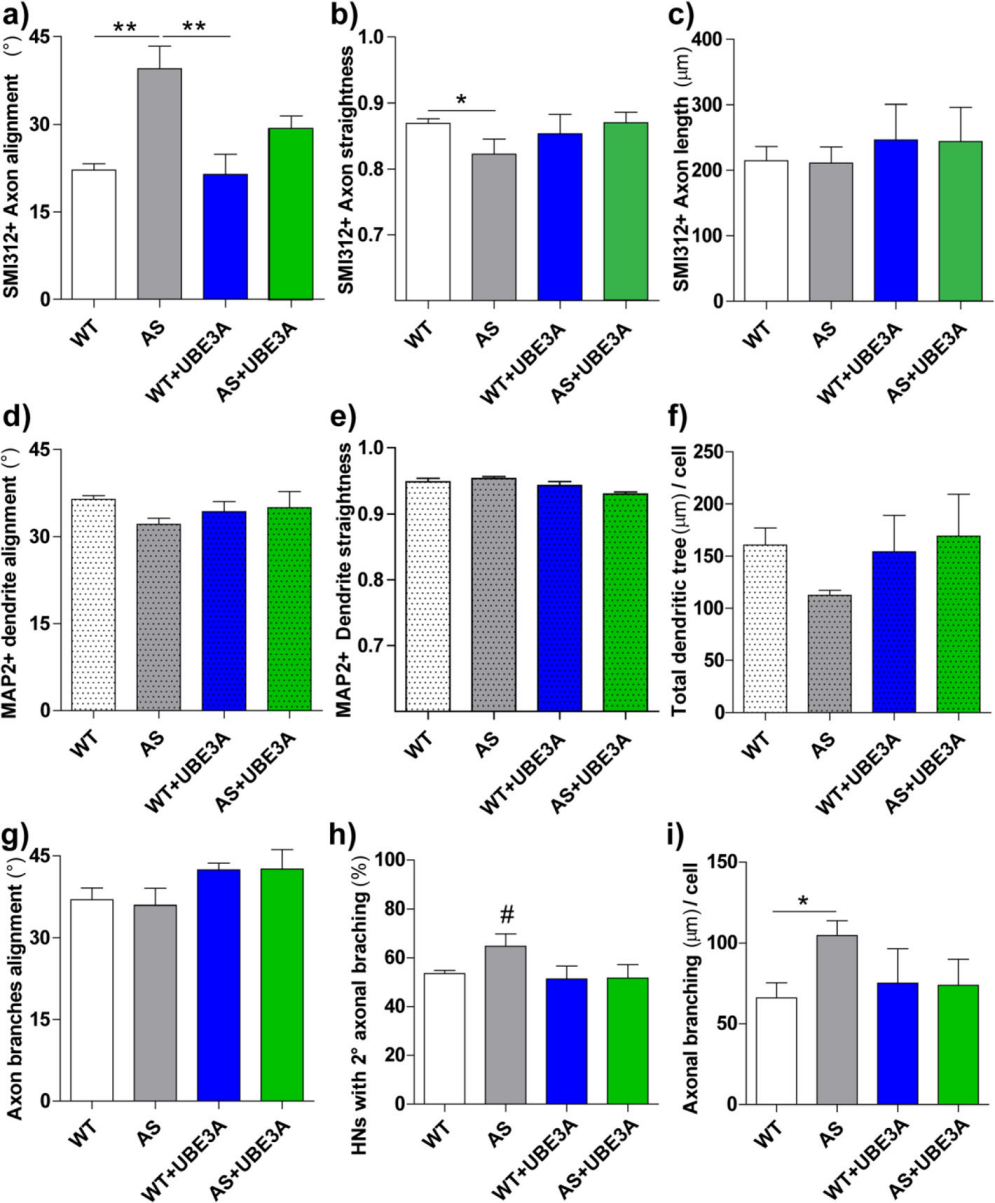
Morphological analysis of WT, AS, WT+UBE3A, and AS+UBE3A neurons (DIV4) cultured on GRs. **a**–**c** Axonal morphological analysis: axon alignment to GR pattern direction (°) (**a**), straightness (**b**), and mean length (μm) (**c**); **a** **P < 0.01 AS vs. WT and vs. WT+UBE3A, Bonferroni’s test; **b** *P < 0.05 WT vs. AS, Bonferroni’s selected test. **d**–**f** Dendritic network morphological parameters: dendrite alignment to GRs (°) (**d**), straightness (**e**), and total dendritic tree length (μm) (**f**); in **f**, ^#^*P* = 0.05 WT vs. AS, Student’ *t* test. **g**–**i** Axonal secondary branching analysis: axon secondary branches alignment to GRs (°) (**g**), the percentage of neurons with branches in the axon (over the total number of neurons analyzed) (**h**), and the amount of axonal branches/neuron (in μm) (**i**); **h** ^#^*P* < 0.05 WT vs. AS, Student’ *t* test; **i** *P < 0.05 AS vs. WT Bonferroni’s selected test. *N* ≥ 4, at least 10 HNs for samples

In order to investigate the role of UBE3A in neuronal contact guidance and to explore neuronal morphological aspects relevant for its imbalance in AS and Dup15q autism, we exploited micrograting (GR) substrates with a grooved pattern of 1-μm ridge, 1-μm groove, and 500-nm depth (Fig. 1a, b), which transfer a directional impulse to neuronal cells by contact interaction [5]. WT and AS primary hippocampal neurons (HNs) were set up on GRs and cultured to achieve the establishment of polarity and axonal specification [53]. After 4 days in vitro (DIV), HNs had already developed a long-polarized axon and a set of shorter dendrites [53]. For each HN, axon (chosen as the longest neurite SMI312 positive) and dendrites (chosen as MAP2-positive neurites and segmented according to their branching) were semi-automatically tracked and their morphological parameters—alignment to the GR anisotropic pattern, straightness, and length (see Methods for details)—were measured (Fig. 1c). WT HNs were mainly polarized along the GR substrate (Fig. 1d) while AS HNs were less polarized (Fig. 1e). By specifically addressing the orientation of axons, we noticed that WT axons exhibited efficient contact guidance (i.e*.*, lower average alignment angle), growing along GRs with an average alignment angle of 22.1 ± 1.1° while AS axons showed reduced axonal alignment with higher angle values (39.5 ± 3.9°; *P* < 0.01 WT vs. AS, Bonferroni test; Fig. 2a). Regarding the axonal path, AS axons showed reduced axonal straightness compared with WT axons (AS, 0.801 ± 0.025; WT, 0.866 ± 0.007; *P* < 0.05, Bonferroni’s test) (Fig. 2b). However, UBE3A loss in HNs did not affect the axonal average length (215 ± 22 μm in WT, 212 ± 26 μm in AS; Fig. 2c). Moreover, to avoid any issue owing to different cell density or to cell-cell interaction in the different samples, we performed an additional test: we set up mixed WT and AS neuronal cultures (i.e., HNs with both genotypes on the same GR sample) on GRs and we analyzed the morphology of WT and AS HNs, by qualitatively identifying them based on differential UBE3A immunostaining (Additional file 1: Figure S1). These results (*n* = 2) confirmed our data, as shown in Additional file 1: Figure S1.

We then studied the effect of overexpressing UBE3A in WT HNs (WT+UBE3A) (Additional file 1: Figure S2a). WT HNs were transfected with a construct expressing both *Ube3a* isoforms 2 and 3 (see details in Additional file 1: Figure S2). Firstly, we assessed the overexpression of UBE3A by immunostaining, confirming the presence of an excess of UBE3A in WT+UBE3A HNs (Additional file 1: Figure S2b). Regarding the guidance performance, WT+UBE3A HN axons polarized along GRs (Fig. 1f) and showed efficient contact guidance similar to WT neurons (Fig. 2a), with an axonal alignment angle of 21.4 ± 3.5° (*P* > 0.05 vs. WT, *P* < 0.001 vs. AS, Bonferroni’s test). Moreover, the increase in UBE3A expression levels did not affect axonal morphological parameters, such as straightness (0.853 ± 0.029) and length (247 ± 54 μm) (Fig. 2b, c).

Having observed that the overexpression of UBE3A does not affect guidance performance, we then tested if UBE3A re-expression in AS neurons could rescue the axonal contact guidance deficit by expressing both UBE3A isoforms 2 and 3 in AS HNs. AS HNs were plated on GRs and then transfected (at early DIV2) with the dual expression vector to reinstate both *Ube3a* isoforms 2 and 3 (AS+UBE3A) (Additional file 1: Figure S2). At DIV4, AS+UBE3A HNs axons grossly polarized along GR pattern (Fig. 1g), despite a partial rescue of the contact guidance performance. In fact, AS+UBE3A showed an intermediate behavior between WT and AS neurons (Fig. 1g), with an axonal alignment angle of 29.3 ± 2.1° and straightness back up to 0.870 ± 0.016 (*P* > 0.05 vs. WT; Fig. 2b). When addressing selectively the orientation of dendrites (MAP2 positive), their development and guidance were not affected by UBE3A loss or increase. The alignment of dendrite segments (Fig. 2d) and their straightness (Fig. 2e) were similar in WT, AS, WT+UBE3A, and AS+UBE3A HNs on GRs. The total length of dendrite tree/cell was similar in WT, WT+UBE3A, and AS+UBE3A HNs, while showing a nominally significant decrease in AS HNs (*P* = 0.05 WT vs. AS, Student *t* test) (Fig. 2f). Due to the occasional presence of secondary branches (SMI312 positive) on the main axon, we investigated also their characteristics (Fig. 2g–i). The number of AS HNs with secondary axonal branching was slightly increased compared with WT neurons (*P* < 0.05 WT vs. AS, Student *t* test) (Fig. 2h). Moreover, the amount of axonal branches/cell was higher in AS HNs (*P* < 0.05 WT vs. AS, Bonferroni’s selected test) (Fig. 2i). However, their alignment was not improved or different with respect to WT neurons (Fig. 2g). WT+UBE3A and AS+UBE3A HNs did not show any difference in secondary axonal branching with respect to WT HNs.

Overall, the total neurite network developed by each cell did not change in different neuronal populations (Additional file 1: Figure S4b). Importantly, the axonal length accounts for more than 50% of the total neurite network of each neuron (see Fig. 2c, f), being primarily responsible for the neuronal polarization direction. Axonal diameter was always around 1.2 μm, with no difference between the HNs genotypes or treatments (details in Additional file 1: Figure S4c). In parallel, on isotropic flat substrates (Flat), axons and dendrites showed a random direction distribution with an average alignment angle around 45° and no difference in morphological aspects between WT and AS HNs (Additional file 1: Figure S4d). Overall, neuronal development was similar on flat and GR substrates, with the only difference in the induction of directional growth on GRs.

We also quantified the neuronal soma features (area, dimensions, and main axis orientation) (Additional file 1: Figure S3). Neuronal somas did not show morphological differences between WT and AS HNs and also after UBE3A reinstatement, and were mainly elongated and oriented along GR direction.

These data demonstrate that, during neuronal polarization, there is a specific deficit in axonal topographical guidance response to directional stimuli in AS neurons, as well as increased axonal branching. Notably, the defective axonal alignment on GRs is specific to loss of UBE3A, as overexpression of UBE3A did not impair axonal guidance. Moreover, UBE3A reinstatement in AS neurons at an early stage of development (DIV2) only partially counteracts this defective process, once already started.

### Growth cones

**Fig. 3:**
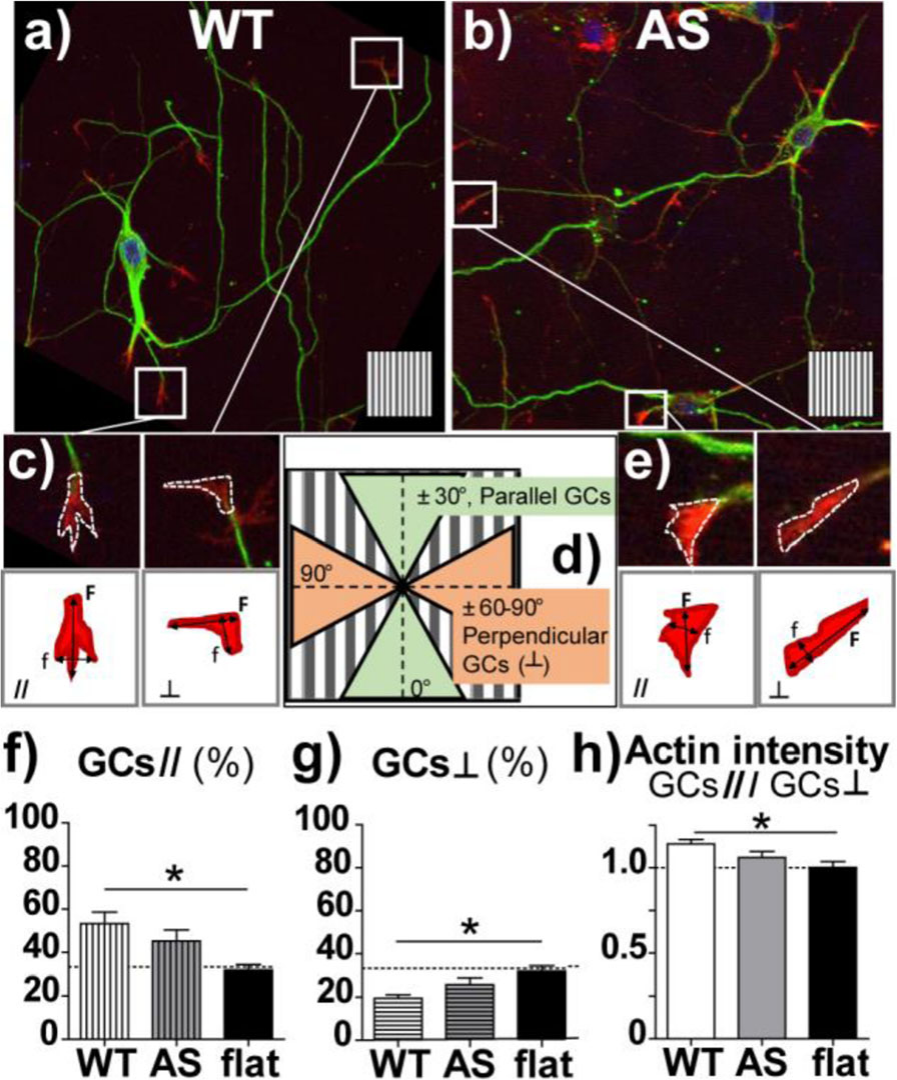
Representative confocal images of growth cones in WT (**a**) and AS (**b**) HNs. Neurons were cultured on GRs and immunostained for tubulin (green), actin fibers (red), and nuclei (blue). The underlying GR pattern is reported as insets; all scale bars = 50 μm. **c**–**e** Growth cones were traced and analyzed by ImageJ: the alignment of their main axis (F) was used to measure their alignment to GR pattern. GCs were categorized in // GCs (if the alignment angle vs. GRs ≤ 30°) or ┴ GCs (if the alignment angle vs. GRs ≥ 60°), as shown in **d**). WT and AS HNs were also cultured on flat substrates and they showed the same random orientation, as expected (therefore they were pooled together). **f** % of GCs aligned (//) to GRs (alignment angle ≤ 30° in respect to the GR direction); **g** % of GCs perpendicular (┴) to GRs (alignment angle ≥ 60° and ≤ 90°); **h** Actin fiber intensity in GCs is reported as the ratio of the actin fiber fluorescent signal in aligned GCs/perpendicular GCs (in a.u.). *P < 0.05 WT vs. Flat, Bonferroni’s test; *n* ≥ 3, at least 35 GCs/sample analyzed

In neurons, growth cones (GCs) play a major role in directing neurite elongation. Therefore, in order to better describe the cellular mechanisms allowing topographical guidance, we investigated GC characteristics in WT and AS HNs exposed to directional GRs. Phalloidin-stained GCs were traced and quantified at the tip of all neurites (Fig. 3). GCs were streamlined and often oriented by GR tracks. WT GCs (53.3 ± 5.4 %) resulted more aligned to the GR pattern in respect to a random orientation (*P* < 0.05 WT vs. Flat; Bonferroni’s test), while AS GCs (45.3 ± 5.1 %) showed an intermediate level of alignment to the GR pattern (*P* > 0.05 AS vs. Flat; Bonferroni’s test) (Fig. 3f); on flat substrates, this percentage reduced to ≈ 33.3% for both WT and AS neuronal populations, corresponding to a random distribution (Flat; Fig. 3f). Accordingly, the amount of GCs developing perpendicularly to GRs (with orientation angle between 60° and 90° vs. GRs) showed exactly the opposite trend (Fig. 3g), with perpendicular GCs at 17.2 ± 3.5% and 23.2 ± 2% in WT and AS, respectively (*P* < 0.01 WT vs. Flat; Bonferroni’s test). GC area was similar in WT (16.5 ± 2.7 μm^2^) and AS neurons (17.8 ± 4.2 μm^2^). Finally, our analysis revealed an increase of the actin fiber content of the aligned GCs (//) with respect to the perpendicular ones (┴), in WT GCs: here, the actin fiber enrichment was significantly enhanced with respect to that measured on flat (*P* < 0.05 WT vs. Flat; Bonferroni’s test), while this effect was not present in AS GCs (Fig. 3h).

Our results suggest that both WT and AS GCs could read and follow the GR topographical stimulus even if with different sensitivity and reinforcement at the level of actin fiber organization.

### Pharmacological tuning

Neuronal cells integrate multiple cues (topographical and chemical) into cytoskeletal signalling that ultimately modulates cell contractility and shaping. We thus hypothesized that the reduced axonal contact guidance and the directional polarization along GRs in AS could be tuned by pharmacologically interfering with the cytoskeleton contractility machinery. WT and AS HNs were cultured on GRs in the presence of nocodazole (Noco; 40 nM), a microtubule depolymerizing agent that activates the RhoA-ROCK-MLC pathway leading to an increase of cell contractility [45, 49], or in the presence of Blebbistatin (Bleb; 25 μM), a myosin-II-contractility inhibiting drug which was shown to interfere with mechanotransduction [4].

**Fig. 4:**
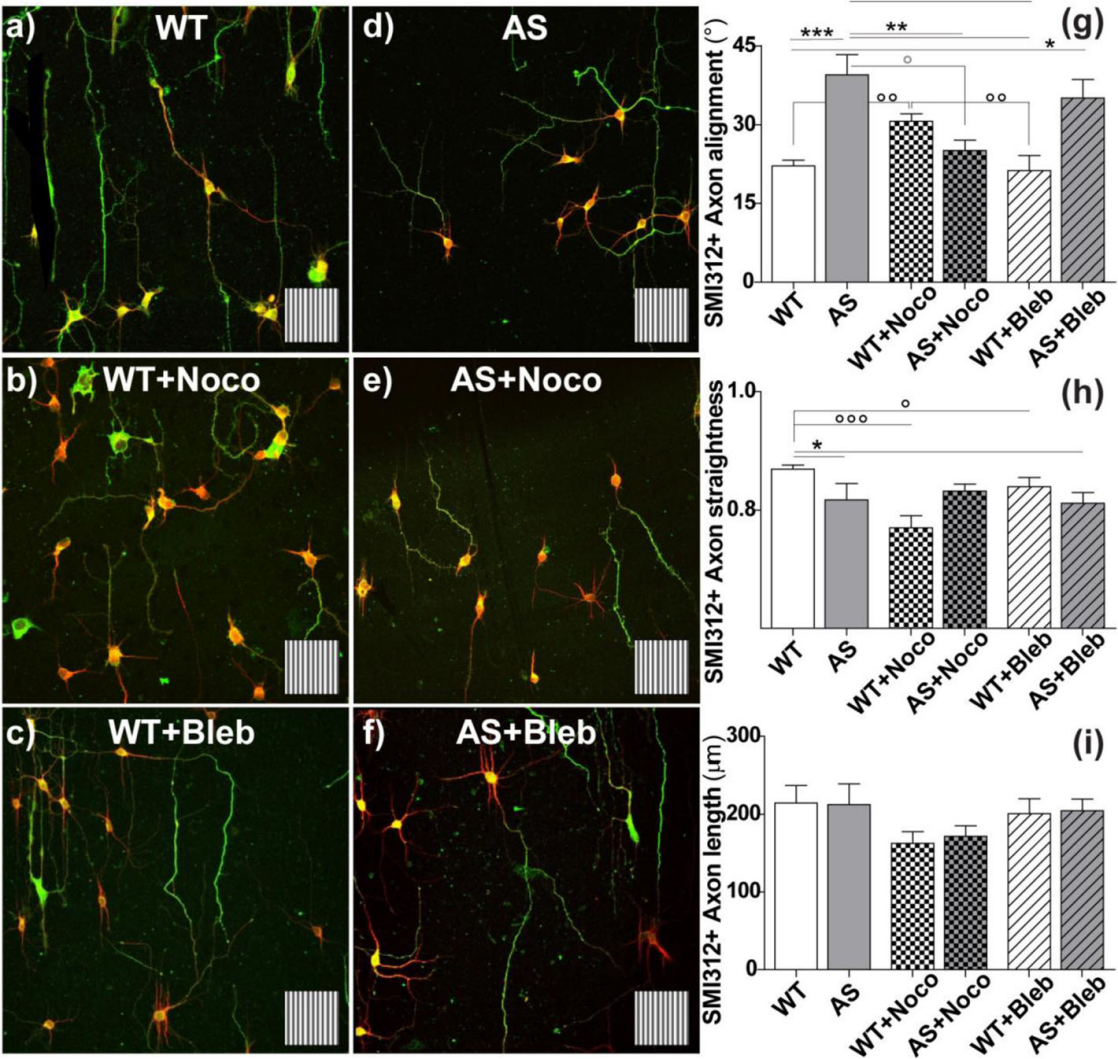
**a–d** Representative confocal images of WT (**a**–**c**) and AS (**d**–**f**) HNs (DIV4), cultured under control conditions (**a**, **d**), or treated with nocodazole 40 nM (**b**, **e**) or with blebbistatin 25 μM (**c**, **f**). Neurons were cultured on GRs, treated with drugs from early DIV2, and immunostained for SMI312 (axonal marker, green) and MAP2 (dendrite marker, red) at DIV4. The underlying GR pattern is reported as insets; inset side = 60 μm. **g**–**i** Axonal morphological parameters of WT (white columns) and AS (grey columns) HNs on GRs in the presence of Noco (squared columns) and Bleb (striped columns): axon alignment (**g**), straightness (**h**), and mean length (**i**) were calculated per each cell. **g** ***P < 0.001 WT vs. AS, *P < 0.05 WT vs. AS+Noco, **P < 0.01 AS vs. AS+Noco and WT+Bleb, Bonferroni’s test; within WT HNs samples: °°*P* < 0.01 WT vs. WT+Noco and WT+Noco vs. WT+Bleb, Bonferroni’s test; within AS HNs samples: °*P* < 0.05 AS vs. AS+Noco, Bonferroni test. **h** *P < 0.05 WT vs. AS and vs. AS+Bleb, Bonferroni’s selected test; within WT HNs samples: °^/^°°°*P* < 0.05–0.001 WT vs. WT+Noco and WT+Bleb, Bonferroni’s test. i *P* > 0.05, Bonferroni’s test. *N* ≥ 3, at least 15 HNs for samples

The effect of drugs was assayed on WT and AS neurons, as shown in Fig. 4. Treatment of WT HNs (Fig. 4a–c) with nocodazole (Noco) significantly impaired axonal alignment along GRs (30.7 ± 1.4°) (*P* < 0.01, WT vs. WT+Noco and WT+Bleb vs. WT+Noco; Fig. 4g) while, surprisingly, blebbistatin (Bleb) did not show any effect (21.3 ± 2.9°) (Fig. 4g). Axonal straightness of WT HNs was negatively affected by both drugs, in particular by Noco (*P* < 0.01 WT vs. WT+Noco, *P* < 0.05 WT vs. WT+Bleb) (Fig. 4h), which may explain the slight reduction of axonal alignment (i.e., increased axon alignment angle) in the case of WT+Noco. In contrast, AS HNs (Fig. 4d–f) showed improved axon alignment (i.e., reduction of the alignment angle values) in the presence of Noco (25.1 ± 2°; *P* < 0.01, AS+Noco vs. AS; Fig. 4g). Importantly, Noco could restore AS axonal development along GRs to WT level (*P* > 0.05, AS+Noco vs. WT) (Fig. 4e, g). On the contrary, Bleb treatment had no effect on the neuronal response to GRs (35.1 ± 3.4°; *P* < 0.05 WT vs. AS+Bleb; Fig. 4g). Both drug treatments had no overt effect on the straightness of AS axons (*P* < 0.05, WT vs. AS and vs. AS+Bleb, Bonferroni’s selected tests; Fig. 4h), in fact, a slight improvement upon Noco treatment was observed (*P* > 0.05, WT vs. AS+Noco, Bonferroni’s selected tests; Fig. 4h). Finally, the drugs did not impact axonal length, both in WT and AS neurons (*P* > 0.05; Fig. 4i).

Noco and Bleb treatment had no major effects on dendrite development, both at level of alignment to GRs and straightness (Additional file 1: Figure S5a, b). However, Bleb showed positive trophic effects on the elongation of the total dendritic tree developed by each cell compared with the control and +Noco conditions, for both WT and AS HNs (*P* < 0.01 WT+Bleb vs. WT and vs. WT+Noco; *P* < 0.01 AS+Bleb vs. AS and vs. AS+Noco) (Additional file 1: Figure S5c). Regarding the axonal secondary branches, their alignment to GRs was similar to dendrites and was not changed by either drug (Additional file 1: Figure S5d). However, treatment of WT HNs with Noco reduced the number of neurons with secondary axonal branching (*P* < 0.05 WT vs. WT+Noco; Additional file 1: Figure S5e). Noco had the same effect on AS HNs, where it reduced the increase of secondary axonal branching, both at the level of the number of neurons with 2° axonal branching (*P* < 0.05 AS vs. AS+Noco; Additional file 1: Figure S5e) and of the axonal branching length/ cell (*P* < 0.01 AS+Noco vs. AS. and AS+Bleb; Additional file 1: Figure S5f).

Overall, in AS neurons, the axonal contact guidance and alignment along the GR pattern was improved by a low dose of Noco (40 nM). We found that AS+Noco axons recover the ability to follow the topography in terms of track alignment, even if their straightness is still not optimal. Noco also normalized the secondary axonal branching in AS neurons, thus likely thereby improving the axonal guidance and development along GRs. Conversely, Noco had an opposite effect on WT axonal development, thus impairing their guidance along GRs, but not affecting axonal branching. In fact, Noco treatment impaired WT axonal alignment by reducing the straight development of axons. Finally, Bleb had no effects on axonal or dendritic directional growth, for WT and AS HNs as well, while it increased dendritic development.

### Focal Adhesion pathway and FA: cytoskeleton link

**Fig. 5:**
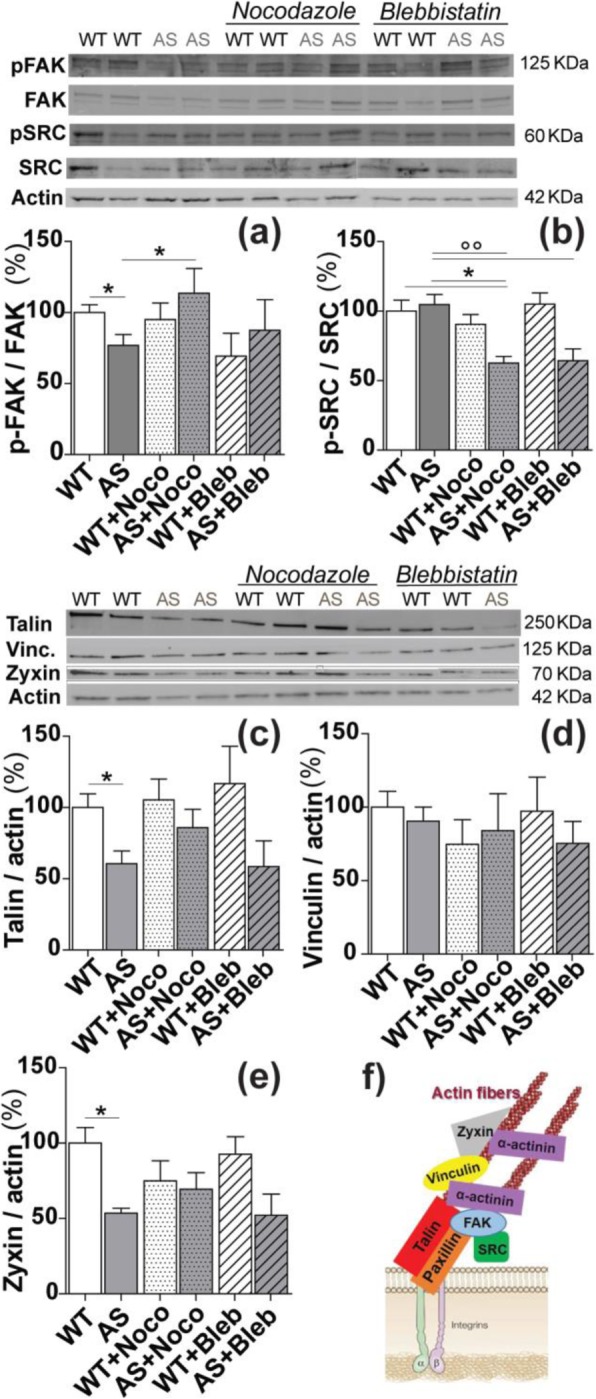
Activation of FA pathway in HNs. Representative western blot panels and blot analysis of phospho-FAK/ FAK (**a**), phospho-SRC/ SRC (**b**), talin (**c**), vinculin (**d**), and zyxin (**e**) levels, in WT (white) and AS (grey) HNs in control, Noco-treated (dotted) and Bleb-treated (striped columns) conditions. Results are reported in % in respect to WT levels: **a** *P < 0.05 WT vs*.* AS and AS vs. AS+Noco, Bonferroni’s selected pair test; **b** *P < 0.05 AS+Noco vs. WT and vs*.* AS, Bonferroni’s test; within AS HNs: °°*P* < 0.01 AS vs. AS+Noco and vs*.* AS+Bleb, Bonferroni’s test; **c** *P < 0.05 WT vs*.* AS, Bonferroni’s selected pair test; **e** *P < 0.05 WT vs*.* AS; Bonferroni’s test; **f** scheme of FA cluster and intracellular signaling. *N* ≥ 5 for control conditions, *n* ≥ 3 for treated conditions

FAs integrate topographical information into cytoskeletal signalling that ultimately modulates cell contractility and shaping [45]. We previously showed that FA activation—at the level of FAK phosphorylation—is impaired in AS HNs [5]. Here, we tested the effect of Noco and Bleb on FAs at the molecular level, on the activation of FAK and SRC and on the level of the scaffold FA proteins talin, vinculin, and zyxin (Fig. 5). WT and AS HNs were cultured on standard plates, treated with or without Noco and Bleb, and lysed at DIV4. Western blot results confirmed the impaired activation of FAK in AS HNs (*P* < 0.05 WT vs. AS, Bonferroni’s selected test; Fig. 5a). Importantly, Noco exposure restored FAK activation to WT control levels (*P* < 0.05 AS vs. AS+Noco, Bonferroni’s selected test; Fig. 5a). The activation of SRC was not affected in AS compared with WT neurons (*P* > 0.05 WT vs. AS; Fig. 5b). However, SRC activation was reduced in AS HNs by Noco (*P* < 0.05 AS+Noco vs. WT and vs. AS; Fig. 5b) and Bleb treatment (*P* < 0.05 AS+Bleb vs. AS; Fig. 5b), whereas SRC activation in WT HNs was insensitive to Noco and Bleb treatment. Regarding the FA scaffolding proteins, talin, a protein linking actin fibers to integrin receptors, was reduced in AS neurons (*P* < 0.05 WT vs. AS, Bonferroni’s selected test) and could be restored by Noco treatment (*P* < 0.05 WT vs. AS, and *P* > 0.05 WT vs. AS+Noco), but not significantly by Bleb treatment (Fig. 5c). Vinculin levels were not affected (Fig. 5d). Finally, we measured the total levels of zyxin, which is only present in mature FAs. Zyxin level was reduced down to 50% in AS with respect to WT HNs (*P* < 0.05 WT vs. AS) (Fig. 5e). When AS HNs were exposed to Noco, zyxin levels were slightly rescued and became not statistically different with respect to the WT control condition. In WT HNs, Noco and Bleb treatments did not severely modify the activation of FAK and SRC, nor the levels of talin, vinculin, and zyxin, while AS neurons result more sensitive to the drugs. Overall, these data show that the maturation and the actin-cytoskeleton anchorage of FAs are altered in AS hippocampal neurons and that the addition of Noco leads to a partial restoration of this phenotype.

**Fig. 6:**
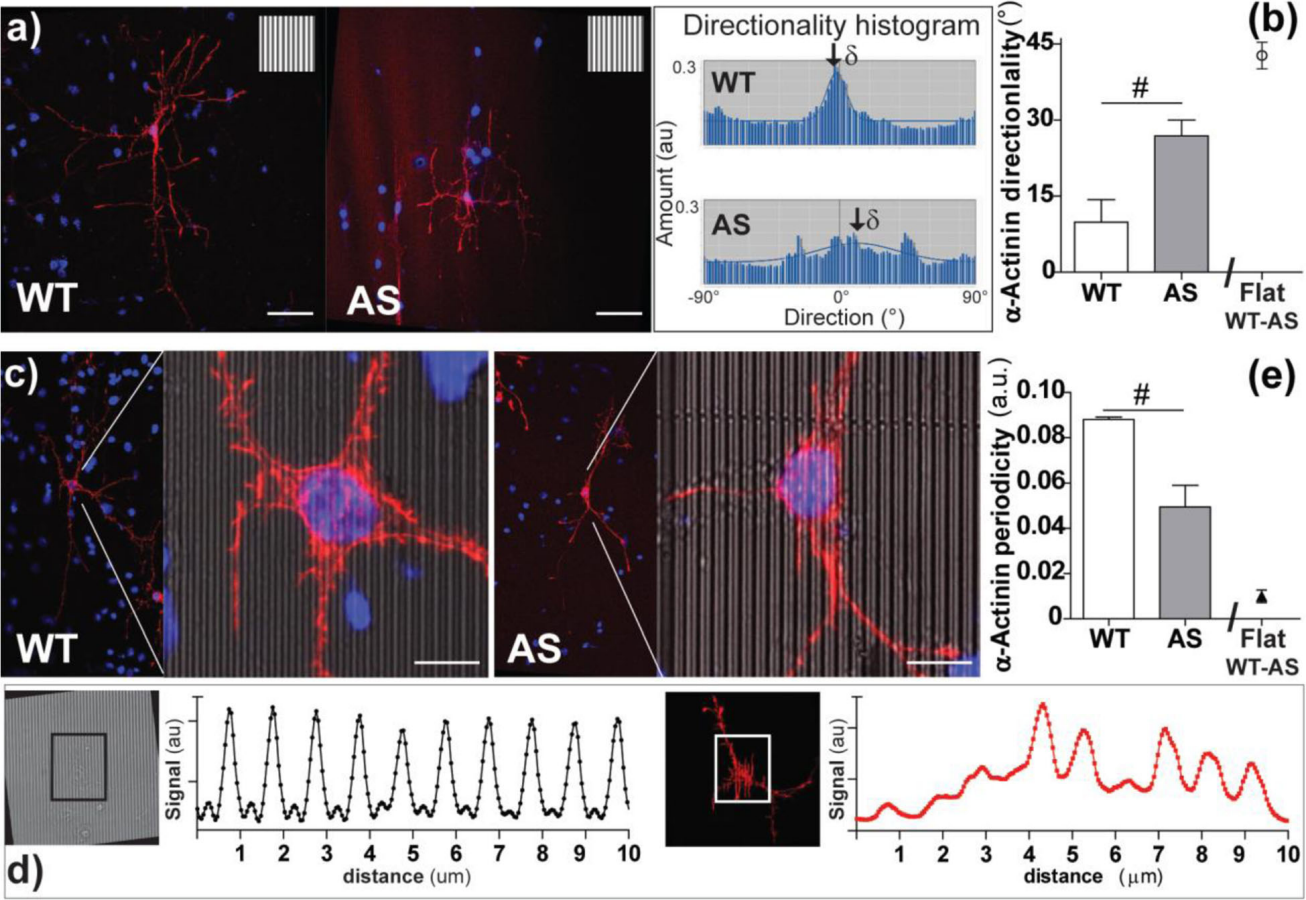
**a** Representative confocal images of WT and AS HNs, transfected with α-actinin-RFP vector and cultured on GRs; the underlying GR pattern is reported as inset, scale bars = 50 μm. Cells have been counterstained with DAPI (blue). **b** Analysis of α-actinin directionality: representative directionality peaked histograms (on the left) are reported for WT and AS neurons, showing the fitted Gaussian curves and the calculation of the α-actinin directionality values (*δ*), normalized to the GR direction (set at 0°). In the graph (on the right), the mean value of α-actinin directionality for HNs cultured on flat standard surfaces (WT and AS pooled together) is also reported, as symbol (*n* = 2 for Flat, reported as mean ± SD); ^#^*P* < 0.05 WT vs*.* AS, Student *t* test. **c** Representative confocal images of WT and AS HNs transfected with α-actinin-RFP and zoomed on soma compartments; scale bars = 10 μm. **d**, **e** Analysis of α-actinin signal periodicity in neuronal soma compartment by Plot profile and FFT analysis: **d** representative examples of Plot profile graphs obtained from the same ROI on the correspondent GRs (left) and α-actinin (right) images, for a WT neuron; **e** the FFT amount of the α-actinin signal is quantified at the GR frequency pick, normalized to the relative RFP intensity, and reported as α-actinin periodicity (in a.u.); the mean value for HNs cultured on flat standard surfaces (WT and AS together) is reported as symbol. *n* = 3 for GRs; *n* = 2 for Flat (here reported mean ± SD). ^#^*P* ≤ 0.05 WT vs*.* AS, Student *t* test

Since α-actinin is a major player in the link between adhesion points and actin fibers [54] and it also interacts with FAK and zyxin, we investigated α-actinin organization in WT and AS neurons. HNs were transfected with α-actinin-RFP and grown on GRs (control experiments were run on flat surfaces). The α-actinin structure appeared qualitatively more organized in WT than in AS HNs (Fig. 6a). At first, in order to quantify the α-actinin structural skeleton, a directionality analysis was performed for each neuron, by exploiting the image fast Fourier transform (FFT) algorithm. Isotropic structures generate a flat histogram, whereas oriented ones give a peaked histogram. These histograms fitted by Gaussian curves returned *directionality* (the center of the Gaussian curve), representing the direction in which the signal is oriented (here normalized to the GR pattern orientation direction). The results showed a higher degree of α-actinin fluorescent signal alignment to GRs in WT HNs (*P* < 0.05 WT vs. AS, Student *t* test) (Fig. 6b). Additionally, to quantify the α-actinin bundle organization with respect to the GRperiodicity, we analyzed α-actinin-RFP signal periodicity in the neuronal soma areas (Fig. 6c) by Plot profile and FFT analysis. We quantified the amplitude of the α-actinin signal at the GR pick frequency (see Methods for details), as readout measurement of the amount of α-actinin bundles developed accordingly to GR pattern (Fig. 6d). The amplitude of the α-actinin signal (normalized to the respective RFP signal) on GRs was higher in WT than in AS cells (*P* = 0.05, Student *t* test), suggesting that α-actinin organization is structured by GRs in WT somas and basal neurites while it is less responsive to them in AS. We also performed control experiments on flat surfaces and in this case the amplitude of the α-actinin signal was not periodic as on GRs, both for WT and AS HNs (Additional file 1: Figure S6).

## Discussion

Here, we investigated the role of UBE3A in contact guidance, with the aim to identify morphological and molecular aspects relevant for neuronal development in AS and ASD/Dup15q. We found that AS HNs have a specific deficit in axonal topographical guidance in response to GR directional stimuli in vitro, and show in parallel increased axonal branching, with more frequent main secondary axonal segments. This deficit is specific to loss of UBE3A, as overexpression of UBE3A has no influence on neuronal guidance. We further demonstrate that AS neurons present impairments in the FA pathway and that their defective axonal guidance can be rescued by low doses of the cytoskeletal drug nocodazole, while only partially by UBE3A protein reinstatement.

Axon formation is the initial step in establishing neuronal polarity [50] and our in vitro results evidence the presence of a specific deficit in the axonal contact guidance of AS HNs in front of a topographical directional stimulus. In line with our present results, Miao et al. [23] demonstrated in vivo that shRNA-mediated downregulation of UBE3A selectively inhibited apical dendrite outgrowth and resulted in impaired polarity both in pyramidal and CA1 neurons (p7). Miao defined *normal dendrite polarity* as a single dendrite within the ± 15° range of orientation toward the pial surface, consistent with previous studies in vivo [55], and similar to in vitro tests [44]. Interestingly the mean axonal alignment of WT HNs on GRs was right around this value (i.e., 22°), while for AS was almost double. However, we did not find a major difference in the dendrite network orientation along the GRs. This might stem from the fact that axonal processes primarily drive cell directional polarization [53] and account for more than 50% of the total neurite length, in vitro. Overall, AS neurons also show an increased number of axonal secondary branches on GRs. It almost seems that AS axons do not know or understand which way they should go, while WT do. Here, Noco normalizes both axonal main directional growth and also its branching. In vivo complex signalling pathways that are activated by extracellular cues regulate the growth and guidance of axon branches but the ultimate target of all these signal transduction pathways is the cytoskeleton (both actin fibers and MTs), which can reorganize by changes in dynamics and polymerization/depolymerization (reviewed in [56]). Our results suggest the presence of defects in cytoskeleton dynamics/ regulation in AS neurons.

Miao et al. [23] further demonstrated that the UBE3A loss–mediated impairment of apical dendrite polarity could be counteracted by coexpressing UBE3A isoform 2, but not isoform 3. However, recently Avagliano Trezza et al. [25] demonstrated the prominent role of UBE3A nuclear isoform 3 in the behavioral and electrophysiological phenotypes of AS mice. For these reasons and for the strong correlation between AS–associated mutations and the loss of Ube3a E3 ligase activity [21], we reinstated both the cytosolic isoform 2 and the nuclear isoform 3 (both catalytically active) to achieve the most promising strategy to rescue the directional guidance of AS neurons. Importantly, reinstatement of UBE3A isoforms 2 and 3 in AS neurons at an early stage of development (DIV2) only partially ameliorated their defective axonal guidance on GRs, once already started (Fig. 2). These “partial” rescue results are in agreement with the demonstrated essential role of UBE3A in the early stages of neurodevelopment [14]. These previous findings were obtained in vivo by using an AS model, which allows for temporally controlled Cre-dependent reinstatement of the maternal *Ube3a* allele [14], and demonstrated that there are distinct temporal windows during which UBE3A restoration can rescue different AS-relevant phenotypes. In fact, most of the AS phenotypes (i.e., epilepsy, autism- and anxiety-related features) appear to be established early and are only rescued when *Ube3a* is reinstated during prenatal or early postnatal development [14]. Likewise, deletion of UBE3A in the mature brain was shown to have little effect [57] emphasizing that UBE3A plays its critical role in early development. Hence, it is possible that the absence of UBE3A during early neuronal development leads to irreversible morphological and wiring defects that later on contribute to the neurological deficits in AS. In line with this scenario, aberrant brain connectivity was revealed at the level of white matter architecture by diffusion tensor imaging in AS patients, pointing out a potential underlying error with axon guidance mechanisms. Microcephaly has been reported in an AS patient [58] and in AS mouse model [15] and a quantitative MRI analysis with voxel-based morphometry recently showed cortical/subcortical grey matter volume loss in AS patients [59], strongly suggesting the presence of developmental abnormalities in AS.

Several data suggest that the proper level of UBE3A is critically important for the normal differentiation of neurons. On our GRs, the defective axonal alignment is due selectively to UBE3A loss, while UBE3A increase (WT+UBE3A) does not impair axonal guidance on GRs. These results are in accordance with previous in vivo reports showing that UBE3A isoform 2 overexpression does not cause any effect on apical dendrite polarity in pyramidal neurons [23]. However, different results have been also reported. The inhibition of terminal dendritic arborization has been reported in the sensory neurons of both Drosophila *Ube3a*-null and *Ube3a*-overexpressing mutants [29]. Moreover, the overexpression of UBE3A induced the loss of dendritic arborization both in vitro in hippocampal neurons (DIV12) and in vivo in the cortex [30]. Overall, UBE3A dosage seems to be crucial during neuronal development, but only its loss impacts negatively on neuronal topographical guidance.

Immature hippocampal neurons (within DIV5) exhibit high levels of UBE3A in GCs [23, 25], and GCs have a primary role in neurite guidance. Our results show that both WT and AS GCs could read and follow the GR topography, even if with different sensitivity and reinforcement at the level of actin fibers organization (Fig. 3). We know that filopodia located at GC tips that are aligned along micropatterned lines are more stable and rich in actin fibers compared with misaligned protrusions [39, 60]. Here, we show that the guidance process is mediated by an anisotropic actin fiber organization at the GC level in WT. Aligned GCs (≤ 15° vs. GRs), indeed, are richer in actin fibers, a condition that may favor (and explain) neuronal growth in the GR direction. This is not the case for AS GCs that show, in particular, a lower ratio of actin fiber content in aligned vs. non-aligned GCs, finally suggesting the presence of deficits in actin fiber formation/stability. Interestingly, Drosophila Ube3a mutants have significantly less filamentous actin than WT larvae, consistent with the identification of actin targets regulated by UBE3A in Drosophila [32]. These defects in GC actin cytoskeletal reorganization likely contribute to the AS impaired guidance on GRs, when GCs are actively in charge of exploring and responding to topographical stimuli, and again point to defects in cytoskeleton responses in AS. In line with our findings, AS mice also showed impaired theta burst stimulation (TBS)–induced actin polymerization, leading to defective synaptic plasticity [31].

Finally, we tested a pharmacological rescue strategy. HNs were cultured on GRs in the presence of a low dose of nocodazole, a microtubule (MT) depolymerizing agent that activates the RhoA-ROCK-MLC pathway, leading to an increase of actin cell contractility [49] and focal adhesion maturation [45]. We previously showed in the PC12 neuronal model that Noco, administered at nanomolar concentration and once that the neurite growth was started (i.e., allowing the MTs stabilization of a major neurite), showed an ameliorative effect on neurite contact guidance response when the GR topographical stimulus is disturbed [45]. Consistently here, the axonal alignment along GRs in AS neurons is improved by a low dose of Noco (i.e., 40 nM), thus restoring the correct directional development at WT levels. Noco is also able to reduce the upregulated secondary axonal branching present in AS neurons. The amelioration of the guidance defects in AS HNs can stem from a Noco-induced increase of actin contractility, in line with other defects found in AS models [31], or of MTs depolymerization. In this framework, Bleb, a myosin-II-contractility-inhibiting drug which was shown to impair mechanotransduction in PC12 neuronal cells [4, 45], had no effect on both WT and AS HNs guidance (Fig. 4), suggesting the presence of additional factors to actin dynamics in this process. We report just a trophic effect on dendritic network length growth upon Bleb treatment, which is in agreement with a previous study [61].

In agreement with previous results showing that FA maturation was favored by Noco treatments [45, 62], we show that Noco rescues some FAs’ molecular effectors that are affected in AS HNs, in particular, at the level of FAK activation (i.e., phosphorylation), talin and, at a little extent, zyxin (Fig. 5). These results confirm the involvement of FAs in AS impaired contact guidance response. Because FA maturation and physical anchorage to cytoskeleton fibers are impaired in AS HNs, our results indicate a possible lower cross-talk between FAs and cytoskeleton fibers in AS neurons. This picture seems to be confirmed by the reduced periodicity of α-actinin bundles organization in AS HNs: in fact, α-actinin exploits an important structural and regulatory roles in cytoskeleton organization and FA maturation [63]. In line with our view, the existence of two distinct pathways for the upregulation of traction forces after Noco-induced MTs depolymerization have been proposed: [1] a Rho-myosin-II-dependent and FAK-independent mechanism, [2] a FAK-dependent and myosin-II-independent pathway [64]. Taken together, the ameliorative effects of Noco on axonal directional growth in AS may stem from its ability to modulate cell traction forces both by increasing actin fiber contractility and by reinforcing FAs, thus activating a feedback crosslink to the actin cytoskeleton.

However, Noco acts primarily on MTs and, in addition to the well-established function of the actin cytoskeleton, local MTs stabilization is a physiological signal specifying axonal/neuronal polarization [65, 66]. In neurons, the loss of polarity correlates with characteristic changes in MTs turnover and consistently, modulation of the MTs stability is sufficient to alter neuronal polarization [65]. Importantly, dendrites differ from axons in patterns of microtubule stability and polymerization during neuronal development [61]. Therefore, our Noco experiments on GRs seem to point out the following: [1] a co-primary role of MTs in AS axonal guidance, [2] a different MTs cytoskeleton stability and reactivity of AS neurons to Noco. Interestingly it has been reported that in neurons of the UBE3A-autism mouse model, the overexpression of UBE3A induces the cleavage of MTs, thus leading to local degeneration and retraction of dendritic branches. Even more interesting, the treatment with 5 nM Taxol, a drug that stabilizes MTs (i.e., therefore with a mechanism opposite to Noco), prevented this UBE3A overexpression-induced morphological changes [30]. These findings indicate that dysregulation in neuronal structural stability is a cellular hallmark in UBE3A-overexpressing autism and suggest a remarkable mirrored situation for AS neurons. We can envision that UBE3A deficiency in AS may cause an excessive stabilization of MTs, leading to contact guidance deficit that can be in turn rescued by low doses of Noco (i.e., which destabilizes MTs at a little extend). In agreement with this, medial ganglionic eminence interneurons exposed to a low (100 nM) concentration of Noco were showed to modify their direction of migration [67]. We also register reduced axonal straightness in AS neurons, likely linked to their lower topographical guidance. However, this aspect is not fully rescued by Noco. On the other side, in WT HNs, the perturbation of the MTs dynamics by Noco is sufficient to alter their axonal growth along GRs, thus resulting in a lower axonal alignment along GRs (i.e., higher alignment angle) and in reduced straightness.

E3 ligases have emerged as key cell-intrinsic regulators of diverse aspects of neuronal morphogenesis and connectivity at distinct temporal phases [13, 68]; however, the specific role of UBE3A in the brain is still unclear. In our view, several UBE3A targets converge on the regulation of cytoskeleton pathways [34]. We already hypothesized, in [5], that the deficits in contact guidance of AS HNs may be linked, directly or indirectly, to deregulations at the level of cytoskeleton dynamics. In our hypothesis, UBE3A could directly regulate upstream one or more mediators of cytoskeleton signaling and its loss (with the consequent accumulation of UBE3A-targets) may lead to impaired axonal topographical guidance during the early phases of neuronal development. The protein p27^Kip1^ (an UBE3A target substrate) [69] stands out now as an interesting candidate. In fact, p27^Kip1^ plays roles in the regulation of the actin and MT cytoskeletons: its loss (i.e., the opposite situation of AS, where p27 should be accumulated) leads to increased RhoA/ROCK-myosin contractility activity, with increased neurite branching and reduced EB3 comets transport and consequently reduced MTs polymerization [70]. Recently, it has been shown that p27^Kip1^ controls the axonal transport and, at the molecular level, does this through the acetylation of MTs [71]. Additionally or alternatively, the impaired contact guidance of AS HNs could also originate from the deregulation of gene expression at the nuclear level. In fact, it has just emerged the important role of the localization of UBE3A [25]: importantly, the clinical AS deficits are mainly due to the loss of the UBE3A isoform 3, with its nuclear localization.

All together, our results indicate the presence of altered cytoskeleton dynamics, at actin and/or MTs levels, in AS HNs in response to topographical stimuli that can be relevant during early neuronal development.

## Limitations

Overall, remodelers of the cytoskeleton, both at the level of actin and MTs dynamics, as well as FAs emerge as important players in UBE3A-mediated processes, and possibly in AS. However, an important limitation of this study is that all data is obtained in vitro and that its relevance in the pathophysiology of AS has to be further established. Moreover, although the molecular players could be exploited as potential therapeutic targets to ameliorate some AS features, given their early role in brain development it appears unlikely that this would result in therapy for AS.

## Conclusions

We exploited microstructured grating substrates allowing the in vitro examination of specific topographical stimuli for neurons. We found that there is a specific deficit in axonal guidance in response to GR directional stimuli in AS HNs, linked to an increased secondary axonal branching. This deficit is specific when UBE3A is lost, while the overexpression of UBE3A has no influence on neuronal guidance. We further demonstrate that AS neurons present impairments in the FA pathway and that their defective contact guidance on GRs can be rescued by low doses of the cytoskeletal drug Noco, while only partial recovery is observed upon UBE3A protein reinstatement.

## Supplementary information

**Additional file 1: Figure S1.** a-b) Representative images of WT (white *) and AS (blue #) HNs, stained for MAP2 as neuronal markers (red) and UBE3A (grey-white), established from mixed WT and AS pups. In the second panel, arrows (white for WT, blue for AS) indicate the axons of selected neurons, visualized by immunostaining for Tau-axonal marker (green), and MAP2-dendrite marker (red). Only fully visible and clearly identifiable (both for genotype and axon specification) neurons have been highlighted here; scale bar = 50 μm. c) Traces and results of axon morphological analysis of WT and AS HNs cultured together, therefore with the exact same density and underlying GRs. Data set analysis of 11 WT and 12 AS axons gave the following results (mean ± SD): alignment angle = 12 ± 10.2 ° for WT, 27.9 ± 19.8 ° for AS; straightness= 0.911 ± 0.090 for WT, 0.865 ± 0.095 for AS. **Figure S2**. a) WT and AS HNs were transfected (at DIV2) with plasmid encoding for UBE3A isoforms 2 and 3 together with tdTomato (Ube3a2/3; YE722), or with an empty plasmid+tdTomato (YE601) as control conditions, processed by immunostaining for MAP2 (*red*) and UBE3A (*grey*) (at DIV4) and imaged as z-stackst (*see Methods*). b) UBE3A expression levels analysis in transfected HNs: the area covered by each Tomato-transfected neuron (*yellow*) was first automatically selected as a *region of interest* (ROI) on the Tomato-positive image (by Threshold and Analyze particles tools in ImageJ); then the ROI was applied to the correspondent UBE3Apositive image and the UBE3A intensity was measured (*Mean grey value*). The UBE3A intensity was normalized to the relative Tomato intensity and reported in arbitrary units (a.u.). UBE3A signal is mostly nuclear in WT neurons while it is basically absent in AS neurons (## *P*<0.01 WT vs. AS, Student t-test). UBE3A signal is highly increased in WT+UBE3A neurons (** *P*<0.01 WT+UBE3A vs. both WT and AS; Tukey’s test), and also in AS+UBE3A (* *P*<0.0 AS+UBE3A vs. both WT and AS; Tukey’s test). Data = mean ± SEM, n = 3 for each condition (at least 5 cells/sample were quantified). **c)** Finally, we quantified the expression of UBE3A isoform 2 and isoform 3 induced by our plasmid YE722 in HEK cells, thanks to their high transfection efficiency (i.e. almost all cells are transfected). HEK cells were transfected with the following plasmids: YE722 (encoding for UBE3A isoforms 2 and 3 +tdTomato), YE702 (isoform3 + tdTomato), YE789 (isoform2 +tdTomato) or YE601 (empty vector +tdTomato). The cells were then processed for westernblotting. Both UBE3A isoform 2 (*higher band*) and isoform 3 (*lower band*) are expressed in the Ube3a2/3 (YE722)-transfected cells. The UBE3A amount is about 67% of isoform 2 and 33 % of isoform 3. **Figure S3.** Soma morphological parameters of WT and AS HNs on GRs substrates, in control conditions and after UBE3A reinstatement: soma *area* (μm^2^); *soma major axis* and *minor axis* for the best-fitted ellipse of the cell soma [52]; *soma alignment angle* (angles were calculated as the absolute value of the difference between the orientation angle of the GRs and of the cell major axis). Neuronal somas have similar dimensions and elongated shape. Data = mean ± SEM, n ≥ 3. **Figure S4.** Neuronal morphological features. **a**) Example of the analysis of axonal morphological features on GRs, in WT and AS neurons transfected with Tomato-empty vector (WT/AS-Tomato transfected; *full symbols*) or not transfected and exposed to 0.2% DMSO in cell medium (WT/AS-NOT transfected; *empty symbols*). We measured and collected the morphometric data separately to check any eventual influence due to the cell transfection or to the DMSO solvent exposure. The results show that there are no differences between the two control conditions, for both WT and AS neurons. Therefore these data were collected together, according to the genotype. Data = mean ± SD, each symbol represents a single sample. b) Total neuritic network mean length (μm) for HNs grown on GRs. c) Axonal mean diameter (μm) for HNs grown on GRs; the axon diameter was measured in its middle part. WT= 1.22 ± 0.08 μm; data = mean ± SEM, n ≥ 3. d) Neuronal morphological parameters of HNs grown on flat standard substrates: here there are no differences between WT and AS HNs (P > 0.05, Student t-test). Data = mean ± SEM, n = 3. **Figure S5.** a-c) Dendritic morphological parameters for WT (*white columns*) and AS (*grey columns*) HNs under different drugs’ treatments - Noco (*dotted columns*) or Bleb (*striped columns*), on GRs: dendrite alignment (a), straightness (b) and total dendritic tree length (μm/cell) (c) were calculated per each cell. */*** *P* < 0.05-0.001, Bonferroni test; # P = 0.05 WT vs. AS, Student t-test. d-f) Axonal secondary branching analysis: axon secondary branches alignment to GRs (°) (d), percentage of neurons with branches in the axon (over the total number of neurons analyzed) (e), and the amount of axonal branches / neuron (in μm) (f); e) * *P* < 0.05 WT vs. AS, Bonferroni selected test; ** *P* < 0.01 WT+Noco vs. AS and AS+Bleb, Bonferroni test; within WT HNs samples: ° *P* < 0.05 WT vs. WT+Noco, Bonferroni test; within AS HNs samples: ° *P* < 0.05 AS vs. AS+Noco, Bonferroni test; f) *** *P* < 0.001 WT+Noco vs. AS, ** *P* < 0.01 WT+Noco vs. WT+Bleb, */° *P* < 0.05 WT+Noco vs. WT+Bleb, Bonferroni test; */°° *P* < 0.05-0.01 AS vs. AS+Noco, ° *P* <0.05 AS+Noco vs. AS+Bleb, Bonferroni test. Figure S6. Representative confocal images of WT (*left*) and AS (*right*) HNs, transfected with α-actinin-RFP vector and cultured on standard coverslips; scale bar= 20 μm.

## Data Availability

The datasets used and/or analyzed during the current study are available from the corresponding author on reasonable request.
