# Supplementary material for: The role of ubiquitin ligase E3A in polarized contact guidance and rescue strategies in UBE3A-deficient hippocampal neurons
Source: Mol Autism. 2019 Nov 29;10:41. doi: 10.1186/s13229-019-0293-1 (PMC6884852; doi:10.1186/s13229-019-0293-1)
Supplement: Supplementary file 1 — Additional file 1: Figure S1. a-b) Representative images of WT (white *) and AS (blue #) HNs, stained for MAP2 as neuronal markers (red) and UBE3A (grey-white), established from mixed WT and AS pups. In the second panel, arrows (white for WT, blue for AS) indicate the axons of selected neurons, visualized by immunostaining for Tau-axonal marker (green), and MAP2-dendrite marker (red). Only fully visible and clearly identifiable (both for genotype and axon specification) neurons have been highlighted here; scale bar = 50 μm. c) Traces and results of axon morphological analysis of WT and AS HNs cultured together, therefore with the exact same density and underlying GRs. Data set analysis of 11 WT and 12 AS axons gave the following results (mean ± SD): alignment angle = 12 ± 10.2 ° for WT, 27.9 ± 19.8 ° for AS; straightness= 0.911 ± 0.090 for WT, 0.865 ± 0.095 for AS. Figure S2. a) WT and AS HNs were transfected (at DIV2) with plasmid encoding for UBE3A isoforms 2 and 3 together with tdTomato (Ube3a2/3; YE722), or with an empty plasmid+tdTomato (YE601) as control conditions, processed by immunostaining for MAP2 (red) and UBE3A (grey) (at DIV4) and imaged as z-stackst (see Methods). b) UBE3A expression levels analysis in transfected HNs: the area covered by each Tomato-transfected neuron (yellow) was first automatically selected as a region of interest (ROI) on the Tomato-positive image (by Threshold and Analyze particles tools in ImageJ); then the ROI was applied to the correspondent UBE3Apositive image and the UBE3A intensity was measured (Mean grey value). The UBE3A intensity was normalized to the relative Tomato intensity and reported in arbitrary units (a.u.). UBE3A signal is mostly nuclear in WT neurons while it is basically absent in AS neurons (## P<0.01 WT vs. AS, Student t-test). UBE3A signal is highly increased in WT+UBE3A neurons (** P<0.01 WT+UBE3A vs. both WT and AS; Tukey’s test), and also in AS+UBE3A (* P<0.0 AS+UBE3A vs. both WT and AS; Tukey’s test). D [file 13229_2019_293_MOESM1_ESM.docx]

***Additional files***


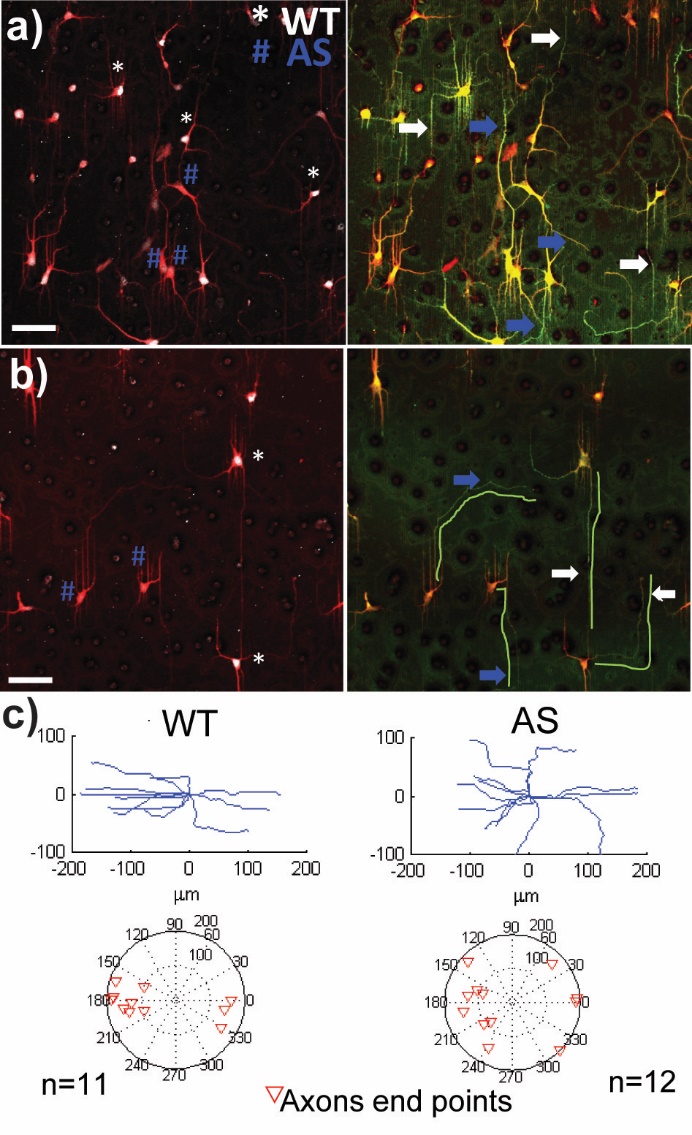


**Figure S1. a-b**) Representative images of WT (*white* *) and AS (*blue* #) HNs, stained for MAP2 as neuronal markers (*red*) and UBE3A (*grey-white*), established from mixed WT and AS pups. In the second panel, arrows (*white* for WT, *blue* for AS) indicate the axons of selected neurons, visualized by immunostaining for Tau-axonal marker (*green*), and MAP2-dendrite marker (*red*). Only fully visible and clearly identifiable (both for genotype and axon specification) neurons have been highlighted here; scale bar = 50 µm. **c)** Traces and results of axon morphological analysis of WT and AS HNs cultured together, therefore with the exact same density and underlying GRs. Data set analysis of 11 WT and 12 AS axons gave the following results (mean ± SD): alignment angle = 12 ± 10.2 ° for WT, 27.9 ± 19.8 ° for AS; straightness= 0.911 ± 0.090 for WT, 0.865 ± 0.095 for AS.


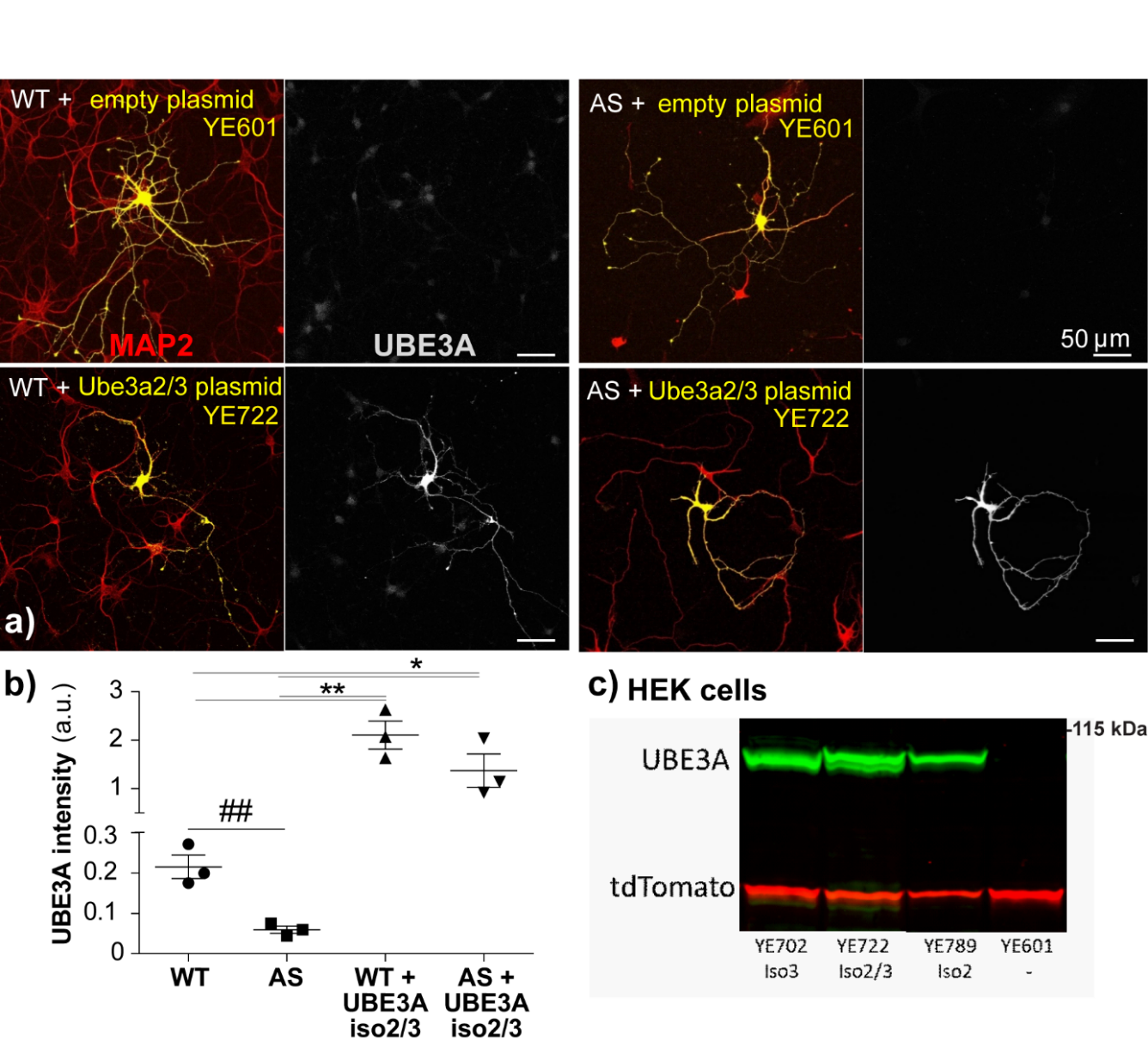


**Figure S2**. UBE3A re-expression levels induced by HNs transfection with plasmid encoding for the UBE3A isoforms 2 and 3 together with Tomato fluorescent protein (tdTomato).

**a)** WT and AS HNs were transfected (at DIV2) with UBE3A isoforms 2 and 3 (Ube3a2/3; named YE722), or with an empty plasmid+tdTomato (named YE601) as control conditions, processed by immunostaining for MAP2 (*red*) and UBE3A (*grey*) (at DIV4) and imaged at confocal microscopy (*see Methods*). **b)** UBE3A levels analysis in transfected HNs: each resulting confocal z-stack (in a) was processed by ImageJ. The area covered by each transfected neuron (*yellow*) was first automatically selected as a *region of interest* (ROI) on the Tomato-positive image (with the “Threshold” and “Analyze particles“ tools); then the ROI was applied to the correspondent UBE3A positive image and the UBE3A intensity was measured by the “Measure” tool (option “*Mean grey value”*). The UBE3A immunostaining intensity was normalized to the relative Tomato intensity and reported in arbitrary units (a.u.). UBE3A signal is mostly nuclear in WT neurons while it is basically absent in AS neurons (## P < 0.01 WT vs. AS, Student t-test). UBE3A signal is highly increased in WT+UBE3A neurons (** P < 0.01 WT+UBE3A vs. both WT and AS; One-Way ANOVA, Tukey’s test, and also in AS+UBE3A (* P < 0.0 AS+UBE3A vs. both WT and AS; Tukey’s test). Data = mean ± SEM, n = 3 independent experiments for each condition (at least 5 cells were quantified for each sample). **c)** Finally, we quantified the expression of both UBE3A isoform 2 and isoform 3 induced by our plasmid. We performed this assay in HEK cells, thanks to their high transfection efficiency (i.e. almost all cells are transfected). HEK cells were transfected with the following plasmids: YE722 (encoding for the UBE3A isoforms 2 and 3 + tdTomato), YE702 (encoding for the isoform3 + tdTomato), YE789 (encoding for the isoform2 + tdTomato) or YE601 (empty vector + tdTomato). The cells were then lysed and processed for western blotting (*see Methods*). Both the UBE3A isoform 2 (*higher band*) and isoform 3 (*lower band*) are expressed in the Ube3a2/3 (YE722)-transfected cells. The UBE3A amount is about 67% of isoform 2 and 33 % of isoform 3.


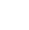

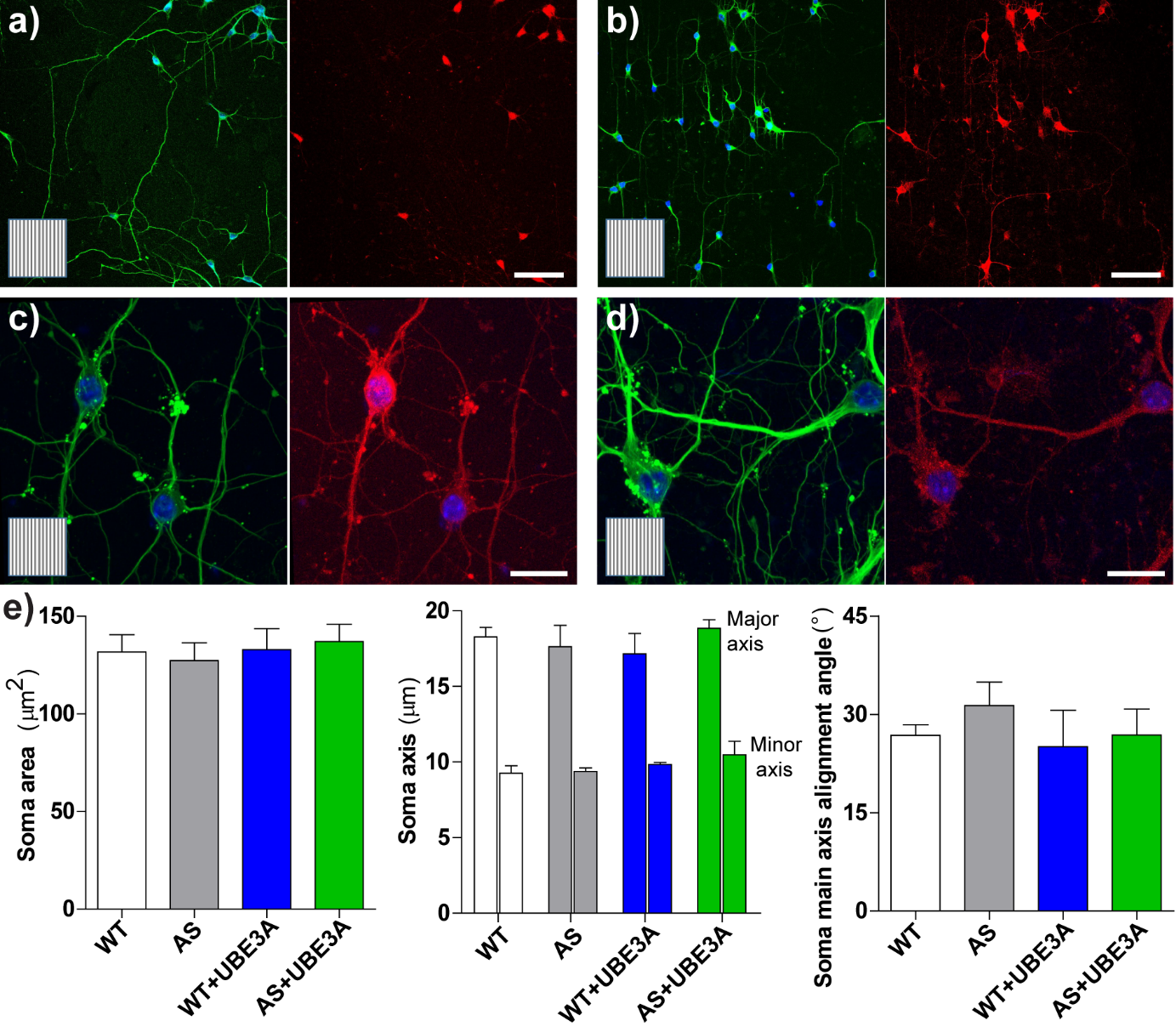


**Figure S3.** Soma morphological parameters of WT and AS HNs on GRs substrates, in control conditions and after UBE3A reinstatement: soma *area* (μm^2^); *soma major axis* and *minor axis* for the best-fitted ellipse of the cell soma (52); *soma alignment angle* (angles were calculated as the absolute value of the difference between the orientation angle of the GRs and of the cell major axis). Neuronal somas have similar dimensions and elongated shape. Data = mean ± SEM, n ≥ 3.


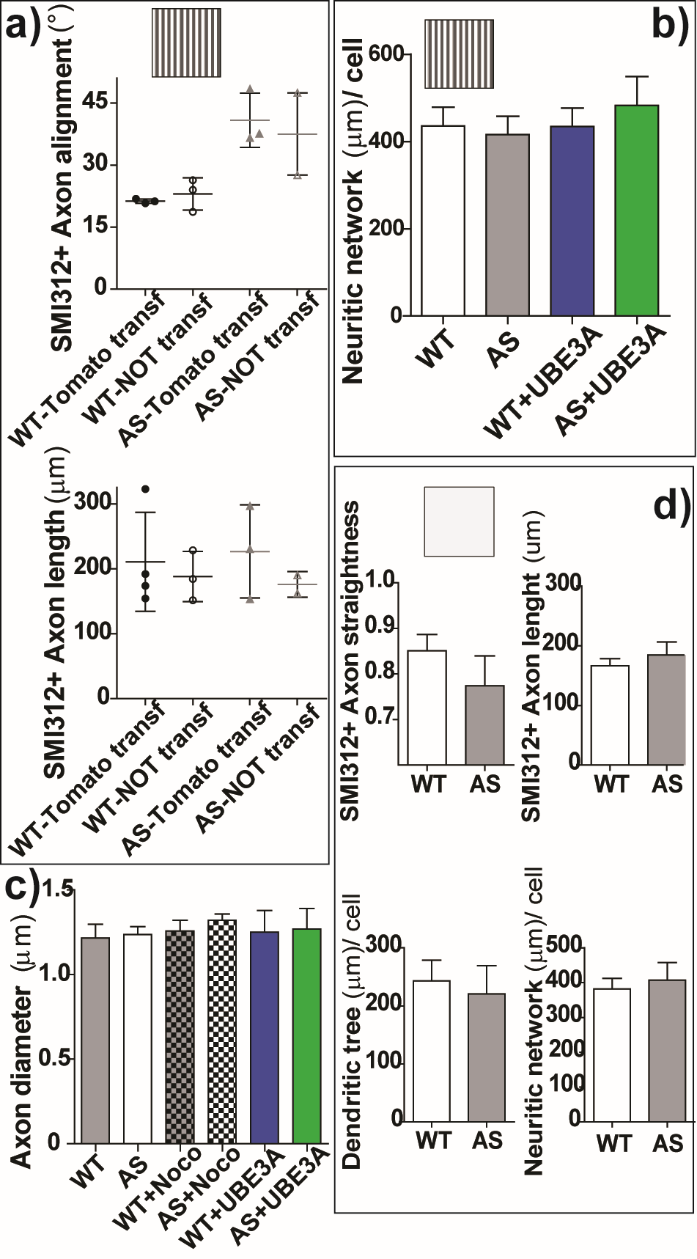


**Figure S4.** Neuronal morphological features. **a**) Example of the analysis of axonal morphological features on GRs, in WT and AS neurons transfected with Tomato-empty vector (WT/AS-Tomato transfected; *full symbols*) or not transfected and exposed to 0.2% DMSO in cell medium (WT/AS-NOT transfected; *empty symbols*). We measured and collected the morphometric data separately to check any eventual influence due to the cell transfection or to the DMSO solvent exposure. The results show that there are no differences between the two control conditions, for both WT and AS neurons. Therefore these data were collected together, according to the genotype. Data = mean ± SD, each symbol represents a single sample. **b)** Total neuritic network mean length (µm) for HNs grown on GRs. **c)** Axonal mean diameter (µm) for HNs grown on GRs; the axon diameter was measured in its middle part. WT= 1.22 ± 0.08 µm; data = mean ± SEM, n ≥ 3. **d)** Neuronal morphological parameters of HNs grown on flat standard substrates: here there are no differences between WT and AS HNs (P > 0.05, Student t-test). Data = mean ± SEM, n = 3.

**
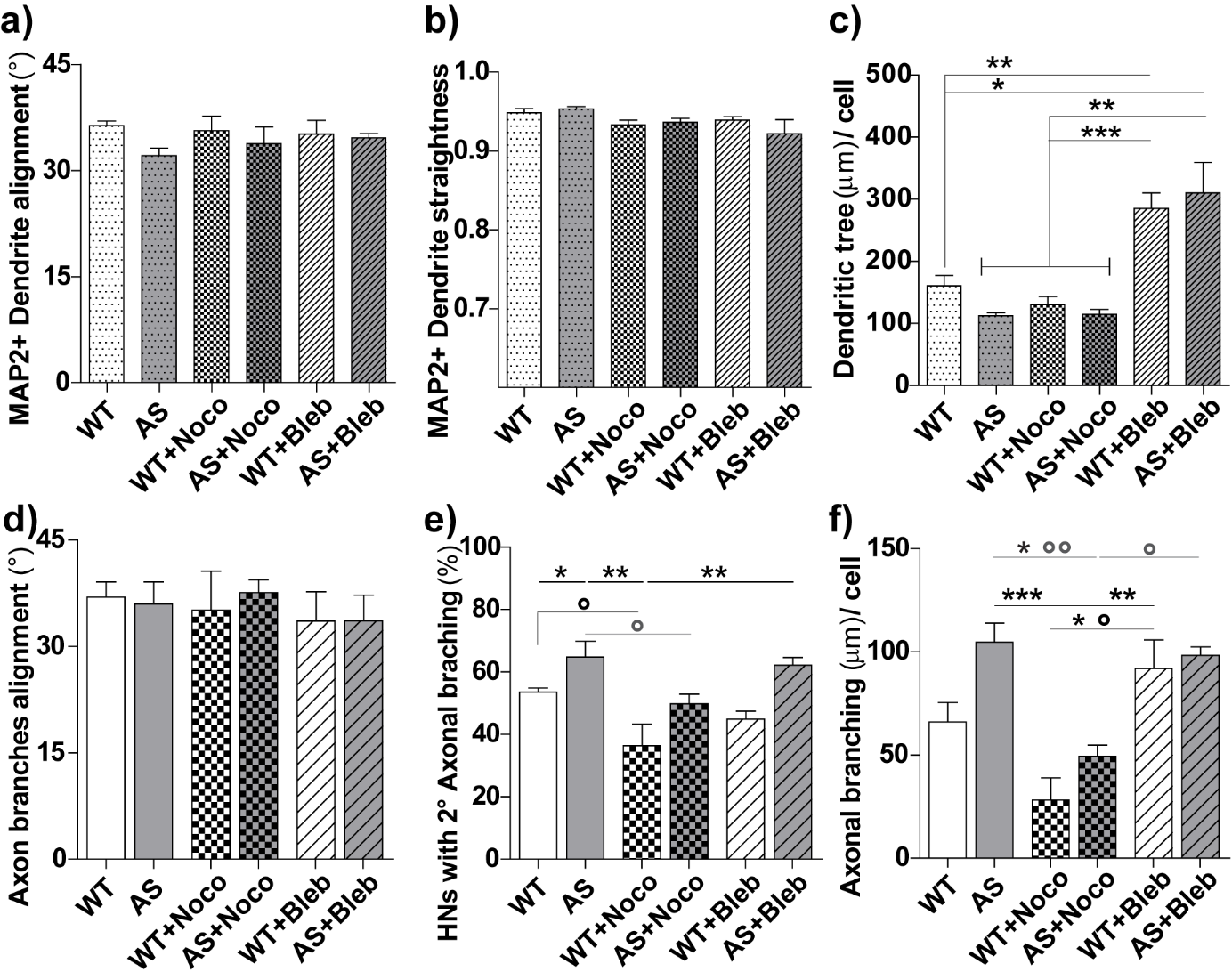
**

**Figure S5. a-c)** Dendritic morphological parameters for WT (*white columns*) and AS (*grey columns*) HNs under different drugs’ treatments - Noco (*dotted columns*) or Bleb (*striped columns*), on GRs: dendrite alignment (**a**), straightness (**b**) and total dendritic tree length (µm/cell) (**c**) were calculated per each cell. */*** P < 0.05-0.001, Bonferroni test; # P = 0.05 WT vs. AS, Student t-test. **d-f**) Axonal secondary branching analysis: axon secondary branches alignment to GRs (°) (**d**), percentage of neurons with branches in the axon (over the total number of neurons analyzed) (**e**), and the amount of axonal branches / neuron (in µm) (**f**); **e**) * P < 0.05 WT vs. AS, Bonferroni selected test; ** P < 0.01 WT+Noco vs. AS and AS+Bleb, Bonferroni test; within WT HNs samples: ° P < 0.05 WT vs. WT+Noco, Bonferroni test; within AS HNs samples: ° P < 0.05 AS vs. AS+Noco, Bonferroni test; **f**) *** P < 0.001 WT+Noco vs. AS, ** P < 0.01 WT+Noco vs. WT+Bleb, */° P<0.05 WT+Noco vs. WT+Bleb, Bonferroni test; */°° P<0.05-0.01 AS vs. AS+Noco, ° P <0.05 AS+Noco vs. AS+Bleb, Bonferroni test.


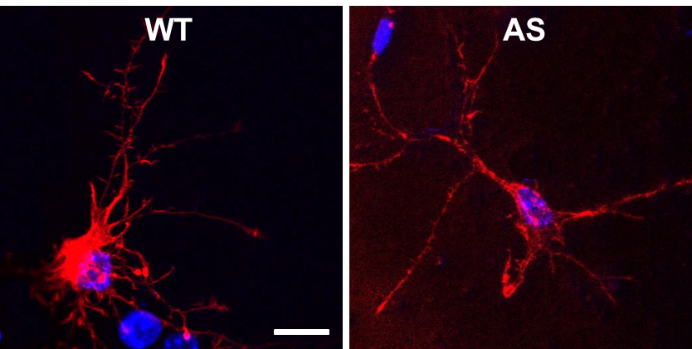


**Figure S6.** Representative confocal images of WT (*left*) and AS (*right*) HNs, transfected with α-actinin-RFP vector and cultured on standard coverslips; scale bar= 20 µm.
